# Emissions and the application of a series of twisted fluorophores with intramolecular weak hydrogen bonds†

**DOI:** 10.1039/c9ra01244c

**Published:** 2019-04-30

**Authors:** Binhong Yu, Danyang Liu, Jinyan Zhang, Zhize Li, Yu-Mo Zhang, Minjie Li, Sean Xiao-An Zhang

**Affiliations:** State Key Laboratory of Supramolecular Structure and Materials, College of Chemistry, Jilin University Changchun 130012 PR China liminjie@jlu.edu.cn seanzhang@jlu.edu.cn; College of Chemistry, Jilin University Changchun 130012 PR China

## Abstract

A series of twisted fluorophores of CEOCH (((2,5-dimethoxy-1,4-phenylene)bis(ethene-2,1,1-triyl))-tetra-benzene) derivatives with intramolecular weak hydrogen bonds (IMWHBs) were synthesized to investigate how different substituents on outer benzenes influence their emissive properties. Because of the twisted structure and weak intermolecular interactions, the emissions of the CEOCH derivatives were intense in the aggregated state but as the flexibility and electronic effect of the substituents changed, their quantum yields (QYs) changed from over 40% to 1% in solution. Based on the adjustable QYs of CEOCHs with different substituents in solutions, a fluorescent sensor for hydrazine with an extremely strong light and dark contrast was obtained *via* the conversion of dicyanovinyl groups to hydrazone groups.

## Abstract 2

A series of twisted fluorophores with intramolecular weak hydrogen bonds were synthesized to investigate how different substituents on outer benzenes influence their emissive properties.

## Graphical abstract

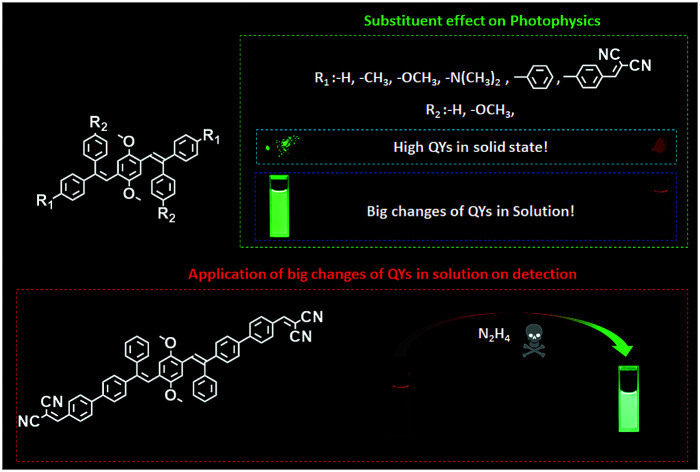

## 1. Introduction

The design and synthesis of efficient organic fluorescent materials have drawn a lot of attention for their widespread and multifunctional applications in OLEDs, sensing, anti-counterfeiting and bio-imaging.^1–5^ Nevertheless, most fluorophores show either aggregation-caused quenching (ACQ) owing to the strong intermolecular forces in the solid state or an aggregation-induced emission (AIE) effect owing to the free motion of the flexible structures in a dilute solution.^6^ There are only a few fluorophores that can exhibit strong emissions irrespective of whether they are in a solid or solution state for weak intermolecular forces and rigid structures.^7–14^ Recently, our group has reported a new twisted fluorophore of ((2,5-dimethoxy-1,4-phenylene)bis(ethene-2,1,1-triyl))-tetra-benzene (CEOCH) with intramolecular weak hydrogen bonds (IMWHBs), which shows strong emissions in both solution and solid states and will definitely extend the applications of fluorophores.^15^ Although there are some reports about weak hydrogen bonds in biological systems or in crystals,^16–29^ there is still a lack of research on the properties and applications of these kinds of twisted molecules with IMWHBs. Thus, it is necessary to develop more twisted fluorophores with IMWHBs, investigate their fascinating fluorescence characteristics and further explore their applications.

**Scheme 1:**
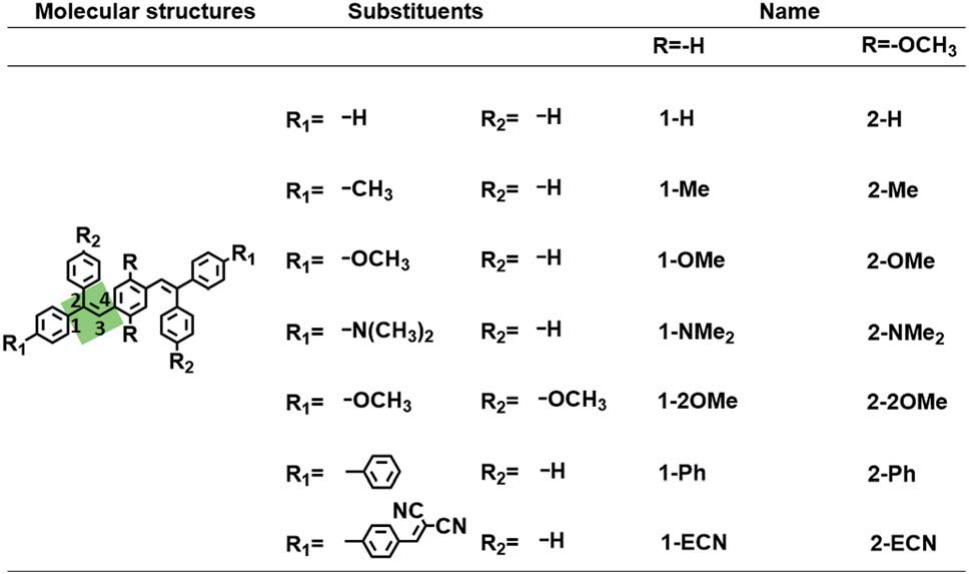
Molecular structures of CEOCHs and their control molecules CEHs.

In this work, a series of twisted CEOCH derivatives, which have differences in their flexibility and electronic properties, have been synthesized, as seen in Scheme 1. 1,4-Bis(2,2-diphenylvinyl)benzene (CEH) derivatives, which have the same conjugated skeleton and substituents as CEOCHs but without a central –OCH_3_ group, were synthesized as the control molecules (Scheme 1).^15^ As the electronic effects of the substituents changed, the emission of the CEOCH derivatives could cover almost the entire visible region. The quantum yields (QYs) of the CEOCH derivatives were all higher than 10% in the aggregated state due to the twisted structures and weak intermolecular interactions. However, their QYs were tuned over 40 times in solution because of the different molecular rigidities resulting from the different flexibilities of the substituents and the different strengths of intramolecular weak hydrogen bonds. Based on the adjustable QYs of CEOCHs with different substituents, an efficient OFF/ON sensor for hydrazine was obtained *via* the conversion of dicyanovinyl groups to hydrazone groups.^30–35^

## 2. Results and discussion

CEOCH, CEH, and their derivatives were prepared by the Wittig reaction and Knoevenagel reaction (Scheme S1†).^15^ Their structures were characterized by ^1^H and ^13^C NMR and mass spectroscopy. According to the multi-peaks of the central –OCH_3_ groups of CEOCH derivatives in their ^1^H NMR spectra, we concluded that there are several stereoisomers in the CEOCH derivatives. Fortunately, the single crystal structure of 2-NMe_2_ (CCDC number: 1906459) was obtained because it has a strong preference towards one configuration, as shown in the NMR spectra; its structure is illustrated in Scheme 1. Other CEOCH derivatives do not have so much preference towards one configuration. However, the calculations on the optimized structures of the different configurations also suggest that the preferred configuration is the same as that of 2-NMe_2_ (Fig. S1–S8 and Table S1†); thus, these structures shown in Scheme 1 were used for further discussion. Further analysis on the peak positions and upfield shifts of ^1^H on the central –OCH_3_ groups with decreasing temperature suggested that IMWHBs exist in all CEOCH derivatives regardless of their configurations (Table S1 and Fig. S3–S6†).

The photophysical properties of CEOCHs and their control molecules were measured to explore how these properties and the intramolecular forces were influenced by different substituents. The detailed absorption and emission peaks as well as the quantum yields (QYs) and lifetimes of CEOCHs and CEHs in THF solutions (10 μM) and solid state are shown in Table 1 and Fig. S9–S16.† First, it was found that the emission peaks of CEOCH and CEH derivatives shift over 100 nm with the substituents in both THF solution (470 nm/655 nm or 456 nm/592 nm) and solid state (477 nm/658 nm or 470 nm/611 nm), covering almost the whole visible spectra. The large shifts in the emission peaks for the CEOCH and CEH derivatives are caused by either the elevated HOMO (highest occupied molecular orbitals) by electro-donating groups or by the descended LUMO (lowest unoccupied molecular orbitals) by electron-withdrawing groups (Fig. S17†).^36,37^ Second, QYs of CEOCHs and CEHs in solid states are all over 10%, which are different from the results of most organic fluorophores whose solid-state emissions are assumed to be strongly quenched due to the increase in the nonradiative decay process by strong intermolecular interactions.^6^ We speculated that the high QYs of CEHs and CEOCHs in solid state are observed because their highly twisted parent structures^15^ have weakened intermolecular forces and restricted free motions in aggregated states (Fig. S1, S8 and S18†).^1,2,6,15^ Third, the absorption peaks and emission peaks of CEOCHs are much more redshifted than those of CEHs due to the introduction of a central –OCH_3_ group.

**Table 1:** Absorption peaks (*λ*^abs^_max_), emission peaks (*λ*^em^_max_), fluorescence lifetimes (*τ*), quantum yields (*ψ*) and corresponding radiative (*k*_r_) and nonradiative decay rates (*k*_nr_) of the CEH and CEOCH derivatives in THF solution (10 μM) and solid state^a^

Molecules	In THF solution						In solid powder				
	*λ* ^abs^_max_ (nm)	*λ* ^em^_max_ (nm)	*τ* (ns)	*ψ* (%)	*k*_r_ (10^8^ s^−1^)	*k*_nr_ (10^8^ s^−1^)	*λ* ^em^_max_ (nm)	*τ* (ns)	*ψ* (%)	*k*_r_ (10^8^ s^−1^)	*k*_nr_ (10^8^ s^−1^)
1-H	352	456	0.55	2.94	0.53	17.64	486	2.28	91.77	4.03	0.361
2-H	389	479	1.29	42.31	3.28	4.47	496	2.29	98.53	4.30	0.0642
1-Me	355	465	0.59	1.87	0.32	16.63	470	2.39	76.06	3.18	1.00
2-Me	390	481	1.35	44.85	3.32	4.08	497	2.30	81.21	3.53	0.817
1-OMe	361	470	0.66	1.22	0.18	14.96	472	1.63	42.48	2.60	3.53
2-OMe	391	487	0.67	11.20	1.67	13.25	488	1.77	71.28	4.03	1.62
1-NMe_2_	392	501	0.89	1.11	0.12	11.11	520	1.20	11.32	0.94	7.39
2-NMe_2_	410	521	0.71	2.35	0.33	13.75	532	1.84	27.98	1.52	3.91
1-2OMe	366	475	0.95	0.73	0.07	10.45	477	1.81	49.94	2.76	2.77
2-2OMe	394	493	0.59	3.34	0.57	16.38	507	1.56	36.64	2.35	4.06
1-Ph	364	475	0.62	14.60	2.35	13.77	481	2.42	74.16	3.15	0.99
2-Ph	395	495	1.47	57.71	3.92	2.87	513	2.14	56.12	2.62	2.05
1-ECN	393	592	2.07	24.17	1.17	3.66	611	18.05	31.70	0.176	0.378
2-ECN	418	655	0.77	1.09	0.14	12.84	658	5.06	11.62	0.230	1.745

a *k*_r_ = *φ*/*τ*; *k*_nr_ = (1 − *φ*)/*τ*.

In addition to the large region of emission peaks in solution, several points can be observed from Table 1: (i) when modified with flexible and electron-donating groups, QYs and *k*_r_ decrease from 1-H, 1-OMe, and 1-2OMe to 1-NMe_2_ or from 2-H, 2-OMe, and 2-2OMe to 2-NMe_2_. Additionally, QYs and *k*_r_ of the CEOCH derivatives are larger than those of the CEH derivatives. (ii) When modified with rigid groups, QYs and *k*_r_ of 1-H, 1-Me and 1-Ph or 2-H, 2-Me and 2-Ph are similar. QYs and *k*_r_ of the CEOCH derivatives are much larger than those of the CEH derivatives.^15^ (iii) When modified with electron-withdrawing groups, QYs and *k*_r_ of 2-ECN are extremely small and the emission of 2-ECN is much weaker than that of 1-ECN. (iv) When the flexibility and electronic effect of the substituents change, QYs of the CEOCH derivatives change over 40 times. Based on the phenomena related to QYs and *k*_r_ of the CEOCH derivatives in solution, we speculate the following points: (i) on increasing molecular motions, non-radiative decay pathways will usually be activated, inducing lower QYs (for example, 2-OMe and 2-NMe_2_). (ii) By comparing CEHs with CEOCHs, when modified with electron-donating groups or rigid groups, the IMWHBs-induced molecular rigidity^15^ is contributed more than the motions of the central –OCH_3_ groups to the emissive intensity. (iii) When the electron-withdrawing groups are introduced, the motions of the central –OCH_3_ group might be dominated and thus, 2-ECN exhibits a much weaker emission than 1-ECN in solution.

**Fig. 1:**
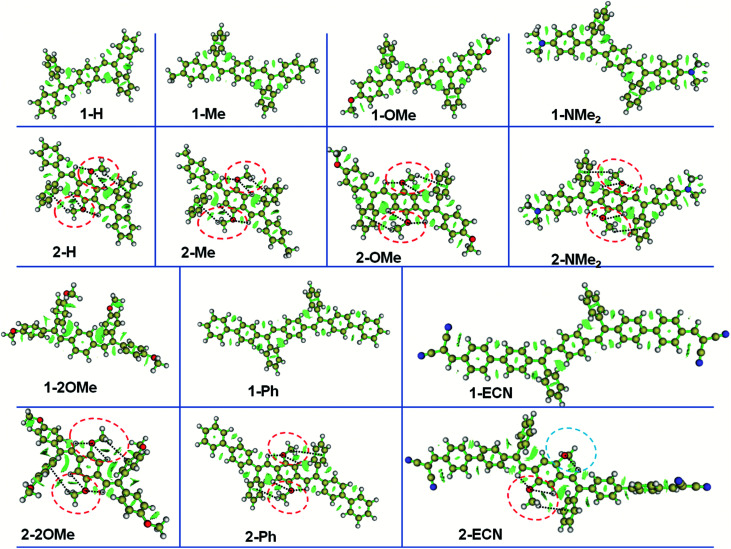
Iso-surfaces of CEHs and CEOCHs, showing intramolecular weak bonds.^40,41^ Yellow, white, red and blue balls represent the C, H, O and N atoms, respectively; green surfaces represent the region of weak interactions; red circles, blue circles and dashed black lines represent the weak interactions related to C⋯O and CH⋯π interactions between the central –OCH_3_ and neighbouring groups (*i.e.*, C=C bonds, central benzenes and outer benzenes).

To reveal the mechanism of adjustable QYs for the CEOCH derivatives in solution, we investigated how the IMWHBs and molecular rigidity are influenced by substituents. First, according to the peak position of ^1^H of central –OCH_3_ and the upfield ^1^H chemical shifts during cooling on the NMR spectra,^28^ we infer that IMWHBs exist in all CEOCH derivatives (Table S1 and Fig. S3–S6†). Then, to further investigate IMWHBs in CEOCHs, the optimized structures of CEOCHs and CEHs in their favourable configurations (as shown in Scheme 1) are calculated based on the B3LYP/6-31G(d,p) level, Gaussian 09 package.^38^ As shown in Fig. S18,† the distances and angles between central –OCH_3_ and neighbouring groups (C=C bonds, central benzenes and outer benzenes) of the optimized CEOCH derivatives are in the region of the CH⋯O and CH⋯π interactions, proving that there are IMWHBs in all CEOCHs.^18,39^ Besides, the iso-surfaces (reduced density gradient) make it possible to directly visualize the weak interactions of CEOCHs.^40^ Through the analysis by the Multiwfn program,^41^ it is observed that weak interactions are also located between the central –OCH_3_ and neighbouring groups (C=C bonds, central benzenes and outer benzenes) of CEOCHs irrespective of the substituents (red circles, blue circles and black lines in Fig. 1), which also proves that there are IMWHBs in all CEOCHs. However, the weak interactions in 2-ECN are much weaker than those in other CEOCHs due to the lack of CH⋯O interactions in one side and less areas of weak bonds (blue circle in Fig. 1 and ESI Movie 1†).^42^ Thus, the above study reveals several points: IMWHBs related to the central –OCH_3_ groups can induce the enhancement of molecular rigidity on the one hand;^15^ on the other hand, central –OCH_3_ groups increase the molecular motions. Therefore, when IMWHBs are strong enough to rigidify the molecular structures and restrict molecular motions, the emission of the CEOCH derivatives is more intense than that of the CEH derivatives (for example, 1-Ph*vs.*2-Ph). When IMWHBs are too weak to rigidify the molecular structures and restrict molecular motions, the emission of the CEOCH derivatives is weaker than that of the CEH derivatives (for example, 2-ECN*vs.*1-ECN).

**Fig. 2:**
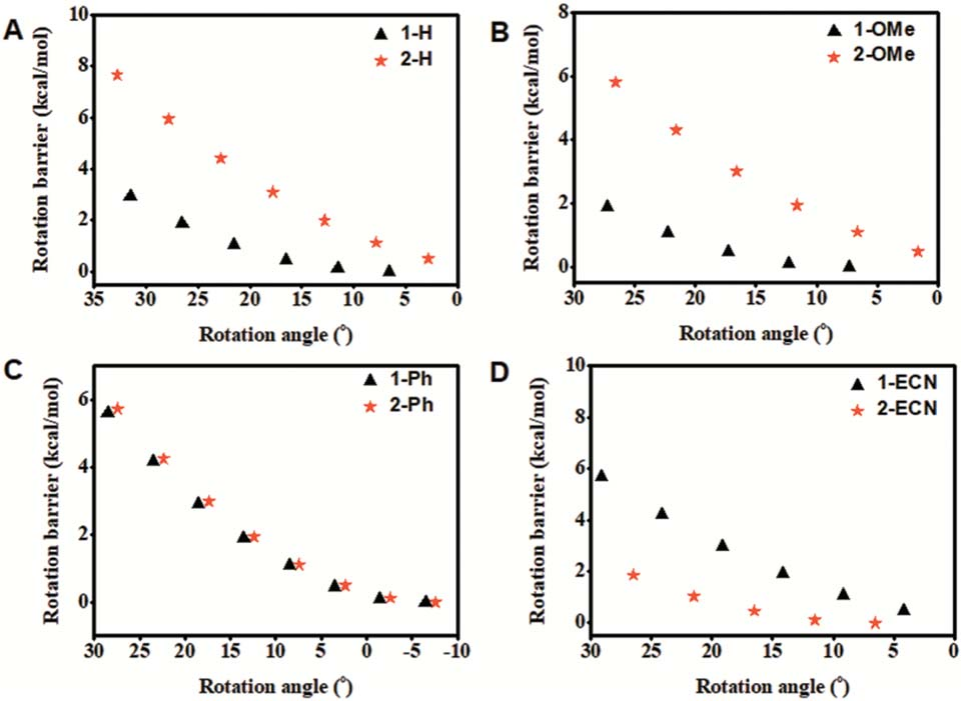
Rotation barrier as a function of the rotation angle (1-2-3-4) in the ground state for isolated 1-H, 2-H, 1-OMe, 2-OMe, 1-Ph, 2-Ph, 1-ECN and 2-ECN.

To further study the relationship between substituents and different QYs of CEOCHs in solution, the rotation barriers that can reflect the molecular rigidity were investigated.^43,44^ As shown in Fig. 2, it can be seen that the rotation barrier of the CEOCH derivatives is larger than that of the control molecules when modified with rigid groups or electron-donating groups, proving that CEOCHs possess more rigid structures than corresponding CEHs. In contrast, when electron-withdrawing groups are introduced, the rotation barrier of 2-ECN is much smaller than that of 1-ECN, suggesting that 2-ECN is more flexible than 1-ECN. Thus, after a comprehensive consideration of the changes in the strength of IMWHBs and the rigidity of the CEOCH derivatives, it is further confirmed that the adjustable QYs of the CEOCH derivatives are caused not only by the flexibility of substituents, but also due to the electron-withdrawing group-induced weakening of IMWHBs and molecular rigidity.

In addition to the study on the rotation barrier, the reorganization energy that can reflect the nonradiative energy loss of fluorophores is also used to evaluate the molecular rigidity.^15,45–48^ Therefore, the DFT calculations of isolated CEOCHs and CEHs in the ground state and excited state were performed based on the B3LYP/6-31G(d,p) level, Gaussian 09 package.^38^ In consideration of the extremely short lifetime and π–π* transitions between the HOMO and LUMO in CEOCHs and CEHs (Table 1 and Fig. S17†), the internal conversion (IC) process from the excited state (S_1_) to the ground state (S_0_) is the main non-radiative decay pathway. In this condition, once the adiabatic excitation energies of CEOCHs and the corresponding CEHs are similar, the key factor that determines the rate of IC is their reorganization energies (*λ*), which reflect the structural relaxation between the ground state and excited state (Scheme S2†).^15,45–48^ Furthermore, the smaller the *λ*, the molecules will be more rigid and possess higher QY.^15,45–48^ Therefore, the adiabatic excitation energies and *λ* of the CEOCH derivatives and the corresponding CEHs are necessary to understand the relationship between the substituents and QYs and to further reveal the function of the central –OCH_3_ group of CEOCHs.

From Table 2 and Fig. S19A,† the following points can be concluded: (i) the adiabatic excitation energies decrease on increasing the electronic effect of the substituents. This suggests that the energy gap between the HOMO and LUMO also decreases and the emission is red-shifted, which agrees with the experimental results. (ii) The adiabatic excitation energies of CEOCHs and the corresponding control derivatives of CEH are quite similar (for example, 2-H*vs.*1-H, 2-OMe*vs.*1-OMe, 2-ECN*vs.*1-ECN), indicating that *λ* is the key factor in the non-radiative pathways of CEOCHs and the corresponding CEHs. Based on *λ* of molecules shown in Table 2 and Fig. S19B,† we can infer the following points: (i) the central –OCH_3_ group plays a large role in rigidifying structures and enhancing the QYs of CEOCHs in their solution when the substituents on the outer benzenes are electron-donating and rigid groups because the *λ* values of 2-H, 2-Me, 2-OMe, 2-NMe_2_, 2-2OMe and 2-Ph are much smaller than that of the corresponding CEHs. (ii) When the outer benzenes of CEOCH are modified with electron-withdrawing groups, the motions of the central –OCH_3_ group of CEOCHs influence the emissive properties more, inducing lower QYs of 2-ECN in solution based on the result that 2-ECN and 1-ECN possess quite similar *λ* values. The calculated results show the influence of the substituents on the emission colour and further reveal the mechanisms of the large difference in the QYs of CEOCHs in solution. The QYs of CEOCHs are not only related to the flexibility of the substituents, but also the adjustable function of central –OCH_3_ in CEOCHs from rigidifying molecular structures to free motions.

**Table 2:** The adiabatic energies (*E*_AD_), reorganization energies (*λ*) and QYs (in THF solution) for isolated CEH and CEOCH derivatives

Molecule	*E*_AD_ (eV)	*λ* (kcal mol^−1^)	QY (%)
1-H	2.73775	18.49237	2.94
2-H	2.60407	14.89887	42.31
1-Me	2.70688	18.49536	1.87
2-Me	2.57911	14.81181	44.85
1-OMe	2.66316	17.29605	1.22
2-OMe	2.54362	14.90451	11.20
1-NMe_2_	2.53618	14.58889	1.11
2-NMe_2_	2.42388	13.50461	2.35
1-2OMe	2.63906	16.37848	0.73
2-2OMe	2.51343	15.55927	3.34
1-Ph	2.60323	16.81437	14.60
2-Ph	2.47583	14.44745	57.71
1-ECN	2.13656	8.36499	24.17
2-ECN	2.02346	8.25129	1.09

**Scheme 2:**
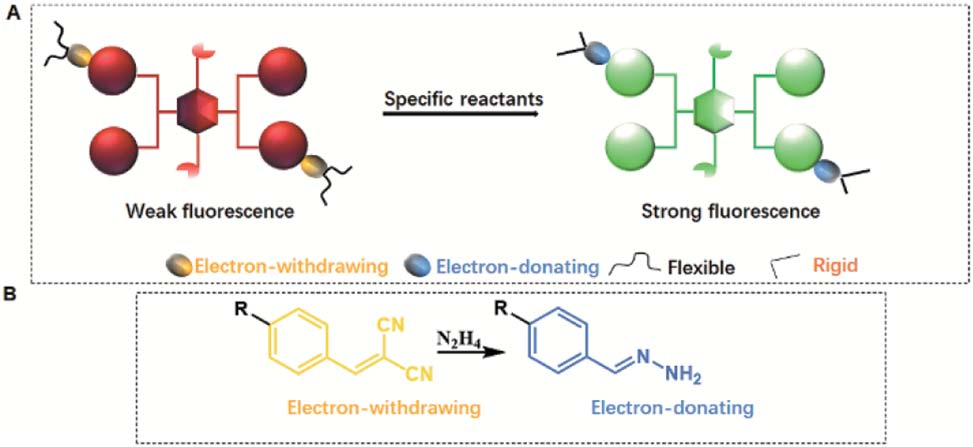
Schematic illustration of re-enhancement of QYs in CEOCHs (A) and the detection of hydrazine (B).

From the above discussion, it is interesting to note that the QYs of CEOCHs can be greatly tuned by changing the rigidity and electronic effect of the substituents on the outer benzenes. Here comes the question of whether this specific phenomenon can be used in detection by utilizing the high luminescent contrast that results from the conversion of the flexible and electron-withdrawing groups on CEOCHs into rigid and electron-donating groups (Scheme 2A). The dicyanovinyl group can react with hydrazine, transforming the electron-withdrawing dicyanovinyl group into a rigid donating hydrazone group (Scheme 2B);^30–35^ thus, the comparisons between 1-ECN and 2-ECN upon addition of hydrazine can not only further clarify our proposed mechanisms of the adjustable QYs of CEOCHs in solution, but also possibly help develop new sensors for detecting toxic hydrazine.^30–35^

**Fig. 3:**
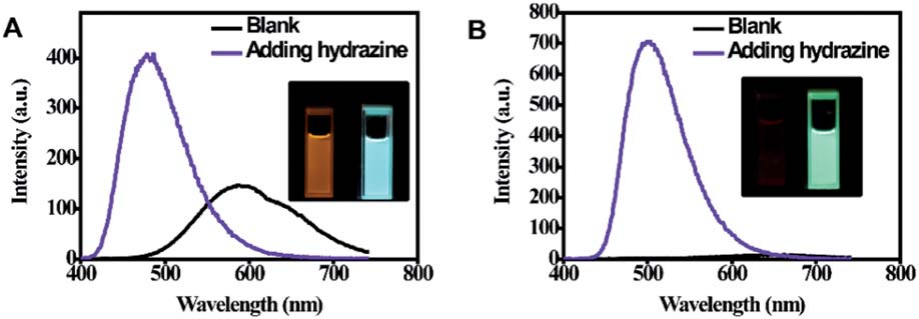
Fluorescence spectra of 1-ECN (A) and 2-ECN (B) in THF solution (10 μM) before and after the addition of hydrazine (4 equiv.). Excitation wavelength: 380 nm. Slits: 1.5, 3. Inset: the corresponding emission images before and after addition of hydrazine.

As shown in Fig. 3, it can be observed that before adding hydrazine, the emission of 2-ECN is weaker than that of 1-ECN. After reacting with hydrazine, the emission is enhanced over hundred times (Fig. S22†) and is much stronger than that of 1-ECN. In addition, the ^1^H NMR titration and mass spectra prove that the enormous emissive enhancement of 2-ECN upon adding hydrazine is indeed caused by the displacement reactions, which convert the dicyanovinyl groups into hydrazone groups (Fig. S20–S21†). Then, through the negligible changes upon the addition of common ions and gradual increment in intensity upon gradual addition of hydrazine to obtain emission spectra, the selectivity and sensitivity of 2-ECN towards hydrazine were clarified (Fig. S22–S24†). Therefore, the manipulation of QYs in CEOCHs could be realized *via* adjusting the substituents on the outer benzene, and a sensor for hydrazine (2-ECN) with high light contrast was obtained based on these results.

## 3. Conclusions

In summary, a series of CEOCH derivatives containing different substituents on outer benzenes with IMWHBs have been synthesized. In their aggregated state, CEOCHs displayed an intense full-colour emission due to their twisted parent structures. In solution state, besides the effect of the flexibility of the substituents on the emission quantum yield, the electronic effect of the substituents could also affect the molecular rigidity by changing the IMWHB strength between central –OCH_3_ and neighbouring groups; it could also affect the emission quantum yield. Furthermore, based on the adjustable QYs of CEOCHs with different substituents in solution, a sensitive, selective and high-luminance contrast sensor (2-ECN) towards hydrazine could be obtained. Overall, the investigation of a series of CEOCHs not only illustrates the relationship among the structure, weak bonds and photophysical properties of twisted molecules, but also promotes new research on utilizing weak interactions in developing new fluorophores and using them in sensing.

## Conflicts of interest

There are no conflicts to declare.

## Supplementary Material

RA-009-C9RA01244C-s001

RA-009-C9RA01244C-s002

RA-009-C9RA01244C-s003
