# Supplementary material for: Emissions and the application of a series of twisted fluorophores with intramolecular weak hydrogen bonds
Source: RSC Adv. 2019 Apr 30;9(23):13214–9. doi: 10.1039/c9ra01244c (PMC9063768; doi:10.1039/c9ra01244c)
Supplement: RA-009-C9RA01244C-s002 [file RA-009-C9RA01244C-s002.pdf]

Electronic Supplementary Material (ESI) for RSC Advances.
This journal is © The Royal Society of Chemistry 2019

Supporting Information

Emissions and the application of a series of twisted fluorophores with intramolecular weak hydrogen bonds

Binhong Yu,[ab] Danyang Liu,[ab] Jinyan Zhang,[ab] Zhize Li,[b] Yu-Mo Zhang,[b] Minjie Li, *[ab] and Sean Xiao-An Zhang*[ab]

[a.] State Key Laboratory of Supramolecular Structure and Materials, College of Chemistry, Jilin University, Changchun 130012, P. R. China

E-mail: liminjie@jlu.edu.cn, seanzhang@jlu.edu.cn.

[b.] College of Chemistry, Jilin University, Changchun 130012, P. R. China

Table of Contents

Instrument and materials

The Preparation and characterization

Analysis on the configurations of CEOCH derivatives

The photophysical properties of CEOCH and CEH derivatives

Calculations and the existence of IMWHBs in CEOCH derivatives

Hydrazine detection

^{1}H and ^{13}C NMR spectra

References

Instrument

^{1}H NMR (500MHz) spectra and ^{13}C NMR (126 MHz) spectra were recorded on a Bruker AVANCE500 using $CDCl_3$ or DMSO-D_6 with tetramethylsilane (TMS) at room temperature. Crystal data were recorded on a Rigaku R-AXIS RAPID. UV-Vis spectra of solutions were measured by an Analytikjena specord 2.10 plus. Fluorescence spectra were recorded by a Shimadzu RF-5301 PC spectrofluorimeter. The fluorescent quantum yield (integration sphere) and life time were measured by Edinburgh Instrument LS920. Infra-red spectra (IR spectra) were recorded on a Brucker VERTEX 80V. Mass was tested by Thermo Fisher ITQ 1100. Images were taken by a Canon camera.

Materials

p-dimethoxybenzene, paraformaldehyde, triethyl phosphite, tetraethyl (1,4-phenylenebis(methylene))bis(phosphonate), , potassium tert-butylate, benzophenone, phenyl(p-tolyl)methanone, (4-methoxyphenyl)(phenyl)methanone, (4-(dimethylamino)phenyl)(phenyl)methanone, bis(4-methoxyphenyl)methanone, [1,1'-biphenyl]-4-yl(phenyl)methanone, 4-bromobenzophenone, malonitrile, potassium acetate, potassium carbonate and [1,1'-bis(diphenylphosphino)-ferrocene]dichloropalladium(II), tetrakis(triphenylphosphine)palladium (0).

Most of the materials were purchased commercially without purification while potassium acetate, potassium carbonate and the solvents, such as THF and CH_2Cl_2 were dried before using were dried before using. The dry THF and DCM were obtained by distillation after refluxing with CaH_2. During testing the photophysical properties of products, all solvents were used directly without purification.

The Preparation of CEH, CECH, CEOH and CEOCH

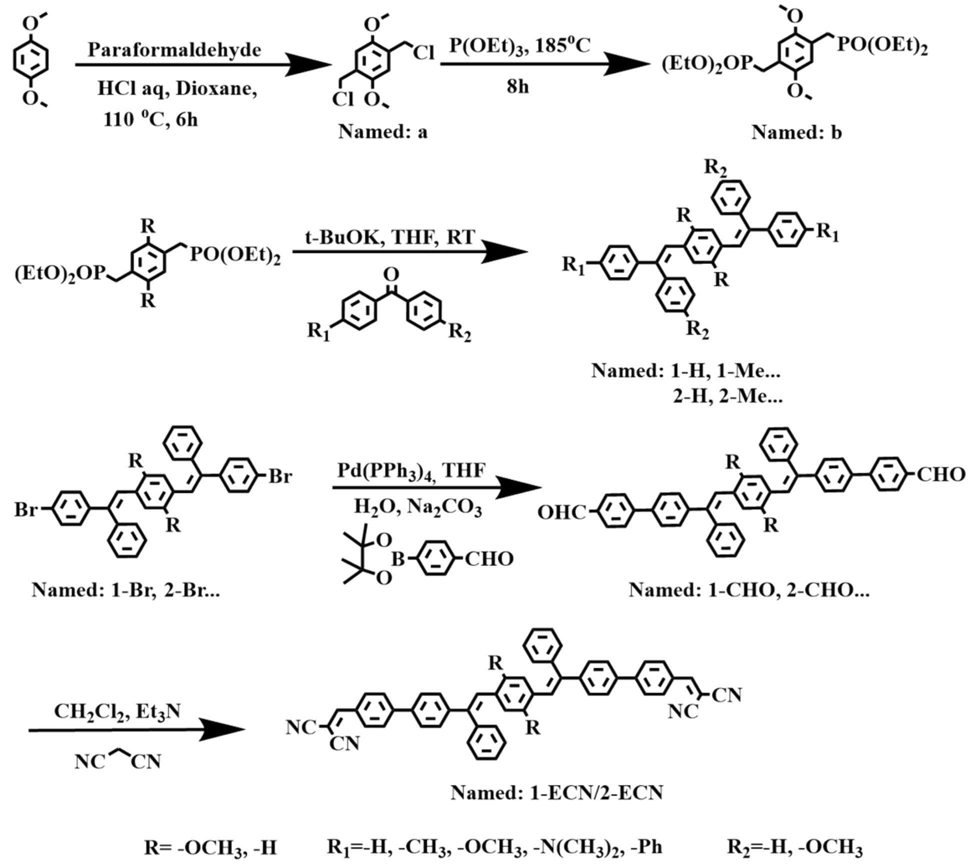

Scheme S1. The syntheses of CEH and CEOCH derivatives.

1,4-Bis(chloromethyl)-2,5-dimethoxybenzene (a). Paraformaldehyde (0.80 g, 26.7 mmol) and p-dimethoxybenzene (1.39 g, 10.1 mmol) were added into round bottom flask with 20 ml 1,4-dioxane and 40 ml concentrated HCl solution. Firstly, the reaction was heated up to 80 °C for few minutes appearing white solid, and then increasing temperature to 110 °C for 6 h. Added large amount of water into reaction mixture and operated filtration to afford light yellow solid. The pure

product (1), white solid was taken from the recrystallization in ethanol. **a (yield: 53%): ^{1}H NMR** (500 MHz, $CDCl_3$) δ = 6.93 (s, 2H), 4.64 (s, 4H), 3.86 (s, 6H).

Tetraethyl ((2,5-dimethoxy-1,4-phenylene)bis(methylene))bis(phosphonate) (b)[1]**.** The product (2) came from the reaction mixing product (1) (1.0g, 4.27 mmol) and triethyl phosphit (1.8 ml, 10.4 mmol) in 185 °C for 8 h. Then, after pouring hexane to reaction system and taking ultrasonic, the white solid appeared and by filtration, the pure product (2) was gained. **b (yield: 72%): ^{1}H NMR** (500 MHz, $CDCl_3$) δ = 6.92 (s, 2H), 4.03 (p, *J*=7.0, 8H), 3.80 (s, 6H), 3.22 (d, *J*=20.3, 4H), 1.24 (t, *J*=7.0, 12H).

Products (substituted CEH and CEOCH)[1]**.** The products were obtained by Witting Reaction (taking CEOCH as an example). Compound (2) (0.876 g, 2.0 mmol) and benzophenone (0.725 g, 3.98 mmol) were added in dry THF (20 ml) in Schlenk flask. Under nitrogen, we added potassium tert-butylate (0.896 g, 8.0 mmol) THF (8ml) solution slowly.[1] This reaction was carried out overnight. Then, adding saturated ammonium chloride aqueous solution into reaction system. The crude product was obtained from the extraction by CH_2Cl_2, and after chromatography using petroleum ether/ CH_2Cl_2, the pure products were obtained. **1-H** (yield: 51%): **^{1}H NMR** (500 MHz, $CDCl_3$) δ 7.35 – 7.23 (m, 17H), 7.17 (dd, *J* = 7.1, 2.2 Hz, 4H), 6.86 (s, 2H), 6.79 (s, 4H); **^{13}C NMR** (126 MHz, $CDCl_3$) δ 143.50, 142.53, 140.40, 135.92, 130.39, 129.20, 128.65, 128.21, 127.91, 127.62, 127.50, 127.47; **GCMS (EI)**: m/z calcd. [M]+ 434.58, found 434.53. **2-H** (yield: 65%): **^{1}H NMR** (500 MHz, $CDCl_3$) δ 7.49 – 7.18 (m, 20H), 7.12 (s, 2H), 6.25 (s, 2H), 3.20 (s, 6H); **^{13}C NMR** (151 MHz, $CDCl_3$) δ 151.02, 143.49, 142.26, 141.01, 130.54, 128.68, 128.09, 127.71, 127.33, 127.24, 125.51, 122.37, 112.30, 55.25; **GCMS (EI)**: m/z calcd. [M]+ 494.63, found 494.65. **1-Me** (yield: 60%): **^{1}H NMR** (500 MHz, $CDCl_3$) δ 7.32 – 7.22 (m, 9H), 7.17 (dd, *J* = 7.3, 2.8 Hz, 4H), 7.13 – 7.02 (m, 6H), 6.85 – 6.74 (m, 6H), 2.40 – 2.28 (m, 6H). **^{13}C NMR** (126 MHz, $CDCl_3$) δ 143.77, 143.73, 142.43, 142.29, 140.66, 140.53, 137.31, 137.06, 135.97, 135.92, 135.89, 135.84, 130.37, 130.33, 130.27, 130.24, 129.29, 129.27, 129.10, 129.07, 128.88, 128.55, 128.11, 127.70, 127.65, 127.63, 127.47, 127.45, 127.37, 127.33, 127.28, 127.12, 127.08, 21.32, 21.31, 21.11. **GCMS (EI)**: m/z calcd. [M]+ 462.64, found 462.11. **2-Me (yield: 65%): ^{1}H NMR** (500 MHz, $CDCl_3$) δ 7.36 – 7.29 (m, 4H), 7.29 – 7.17 (m, 9H), 7.16 – 7.02 (m, 8H), 6.25 (dd, *J* = 11.5, 2.6 Hz, 2H), 3.20 (d, *J* = 11.8 Hz, 6H), 2.34 (d, *J* = 7.9 Hz, 6H). **^{13}C NMR** (126 MHz, $CDCl_3$) δ 150.93, 143.63, 141.15, 140.70, 137.95, 137.14, 136.83, 130.51, 130.43, 129.29, 128.80, 128.61, 128.03, 127.71, 127.58, 127.24, 127.13, 125.53, 122.15, 121.55, 112.29, 55.21, 55.10, 21.17, 21.12. **GCMS (EI)**: m/z calcd. [M]+ 552.69, found 522.12. **1-OMe (yield: 42%): ^{1}H NMR** (500 MHz, $CDCl_3$) δ 7.28 (ddd, *J* = 18.2, 10.1, 6.8 Hz, 9H), 7.23 – 7.13 (m, 4H), 7.09 (dd, *J* = 8.2, 6.1 Hz, 2H), 6.87 – 6.72 (m, 10H), 3.86 – 3.74 (m, 6H). **^{13}C NMR** (126 MHz, $CDCl_3$) δ 159.19, 158.93, 143.88, 142.01, 141.87, 140.57, 136.10, 135.80, 132.55, 131.59, 130.34, 129.12, 129.08, 129.04, 129.00, 128.72, 128.56, 128.11, 127.67, 127.56, 127.39, 127.34, 126.27, 113.97, 113.56, 55.29, 55.18. **GCMS (EI)**: m/z calcd. [M]+ 494.63, found 494.12. **2-OMe (yield: 51%): ^{1}H NMR** (500 MHz, $CDCl_3$) δ 7.39 – 7.18 (m, 13H), 7.14 (dd, *J* = 8.5, 3.6 Hz, 2H), 7.05 (d, *J* = 8.1 Hz, 2H), 6.91 – 6.77 (m, 4H), 6.28 (dd, *J* = 48.9, 6.7 Hz, 2H), 3.86 – 3.72 (m, 6H), 3.31 – 3.14 (m, 6H). **^{13}C NMR** (126 MHz, $CDCl_3$) δ 158.87, 151.01, 143.83, 141.19, 133.13, 131.75, 130.50, 128.85, 128.62, 128.02, 127.80, 127.29, 127.14, 122.09, 120.65, 119.96, 114.02, 113.48, 112.25, 55.29, 55.27, 55.20. **GCMS (EI)**: m/z calcd. [M]+ 554.69, found 554.18. **1-NMe$_2$(yield: 16%): ^{1}H NMR** (500 MHz, $CDCl_3$) δ 7.34 – 7.13 (m, 13H), 7.04 (t, *J* = 9.3 Hz, 2H), 6.94 – 6.83 (m, 2H), 6.80 – 6.58 (m, 8H), 2.96 (d, *J* = 13.5 Hz, 12H). **^{13}C NMR** (126 MHz, $CDCl_3$) δ 149.50, 144.54, 142.46, 142.03, 140.91, 136.24, 136.10, 135.85, 131.44, 130.48, 130.44, 129.10, 129.05, 128.97, 128.91, 128.53, 128.42, 128.05, 127.97, 127.95, 127.28, 127.20, 127.04, 124.86, 112.49, 40.68. **GCMS (EI)**: m/z calcd. [M]+ 520.72, found 520.19. **2-NMe$_2$ (yield: 30%): ^{1}H NMR** (500 MHz, $CDCl_3$) δ 7.33 (t, *J* = 7.4 Hz, 4H), 7.23 (dt, *J* = 18.6, 9.1 Hz, 12H), 7.11 – 6.94 (m, 3H), 6.65 (s, 4H), 6.21 (d, *J* = 10.3 Hz, 2H), 3.24 (d, *J* = 47.7 Hz, 6H), 2.95 (s, 12H). **^{13}C NMR** (126 MHz, $CDCl_3$) δ 151.00, 150.86, 144.28, 142.38, 141.46, 131.52, 130.59, 128.59, 128.00, 127.97, 127.97, 127.95, 127.17, 127.14, 127.05, 127.02, 125.84, 121.45, 112.24, 112.21, 112.12, 77.30, 77.05, 76.79, 55.42, 55.38, 55.28, 55.24, 40.95. **GCMS (EI)**: m/z calcd. [M]+ 580.77, found 580.21. **1-2OMe (yield: 41%): ^{1}H NMR** (500 MHz, $CDCl_3$) δ 7.29 – 7.17 (m, 5H), 7.09 (d, *J* = 8.3 Hz, 4H), 6.82 (dd, *J* = 13.7, 5.2 Hz, 11H), 6.72 (s, 2H), 3.81 (d, *J* = 12.9 Hz, 12H). **^{13}C NMR** (126 MHz, $CDCl_3$) δ 159.22, 158.94, 141.51, 136.56, 135.99, 132.80, 131.63, 129.01, 128.87, 126.05, 113.97, 113.55, 55.33, 55.20. **GCMS (EI)**: m/z calcd. [M]+ 554.69, found 554.29. **2-2OMe (yield: 53%)**: **^{1}H NMR** (500 MHz, $CDCl_3$) δ 7.25 (d, *J* = 5.9 Hz, 5H), 7.14 (d, *J* = 8.6 Hz, 4H), 6.97 (s, 2H), 6.87 (d, J = 8.7 Hz, 4H), 6.82 (d, J = 8.8 Hz, 4H), 6.30 (s, 2H), 3.80 (d, J = 4.7 Hz, 13H), 3.27 (s, 6H). **^{13}C NMR** (126 MHz, $CDCl_3$) δ 159.16, 158.88, 150.95, 141.50, 136.51, 133.33, 131.80, 131.45, 131.00, 128.99, 125.68, 120.40, 114.01, 113.44, 55.32, 55.30. **GCMS (EI)**: m/z calcd. [M]+ 614.74, found 614.17. **1-Ph (yield: 65%): ^{1}H NMR** (500 MHz, $CDCl_3$) δ 7.68 – 7.48 (m, 8H), 7.49 – 7.14 (m, 21H), 6.97 – 6.74 (m, 6H). **^{13}C NMR** (126 MHz, $CDCl_3$) δ 143.58, 142.22, 142.10, 140.70, 140.33, 140.09, 139.42, 136.02, 135.95, 130.94, 130.91, 130.41, 129.27, 128.84, 128.75, 128.27, 128.14, 127.98, 127.85, 127.78, 127.59, 127.35, 127.22, 127.01, 126.98, 126.92. **GCMS (EI)**: m/z calcd. [M]+ 586.78, found 586.10. **2-Ph (yield: 72%): ^{1}H NMR** (500 MHz, $CDCl_3$) δ 7.58 (d, *J* = 7.7 Hz, 6H), 7.51 (d, *J* = 8.3 Hz, 2H), 7.48 – 7.22 (m, 21H), 7.20 (d, *J* = 4.1 Hz, 1H), 7.15 (d, *J* = 1.8 Hz, 1H), 6.37 – 6.21 (m, 2H), 3.21 (d, *J* = 6.2 Hz, 6H). **^{13}C NMR** (126 MHz, $CDCl_3$) δ 151.05, 143.39, 141.98, 140.97, 140.84, 140.15, 140.08, 131.08, 130.57, 128.90, 128.79, 128.18, 128.07, 127.83, 127.47, 127.37, 126.97, 126.82, 125.59, 122.62, 122.31, 118.23, 112.39, 55.26, 55.16. **GCMS (EI)**: m/z calcd. [M]+ 646.83, found 646.10. **1-Br (yield: 53%): ^{1}H NMR** (500 MHz, $CDCl_3$) δ = 7.48 – 7.36 (m, 4H), 7.34 – 7.27 (m, 6H),

7.25 (d, *J*=2.2, 2H), 7.19 – 6.97 (m, 6H), 6.89 – 6.74 (m, 6H). **2-Br (yield: 64%):** ^{1}**H NMR** (500 MHz, $CDCl_3$) δ 7.47 (d, *J* = 7.9 Hz, 2H), 7.43 – 7.27 (m, 10H), 7.19 (dd, *J* = 17.5, 7.9 Hz, 4H), 7.10 (dd, *J* = 11.3, 5.4 Hz, 4H), 6.22 (d, *J* = 5.9 Hz, 2H), 3.23 (dd, *J* = 51.5, 10.3 Hz, 6H).

Products (1-CHO, 2-CHO)[2]**.** Through traditional Suzuki Reaction, the products (1-CHO/1o-CHO) were obtained. Taking CEOCH-CHO as an example, compound (1o-Br) (0.45 g, 0.69 mmol) and 4-(4,4,5,5-tetramethyl-1,3,2-dioxaborolan-2-yl)benzaldehyde (0.32 g, 1.38 mmol) was reacted under the influence of tetrakis(triphenylphosphine) palladium (0) (20 mg) catalyst in THF/water (10 ml/ 2 ml) at 72 ^{o}C for 48 h. After cooling, the reactants were poured into water and then extracted by dichloromethane. The solvent was removed by reduced pressure evaporation. The crude product was purified by chromatography using petroleum ether/ dichloromethane. The green solid was obtained. **1-CHO (yield: 42%):** ^{1}**H NMR** (500 MHz, $CDCl_3$) δ = 10.12 – 9.98 (m, 2H), 7.95 (dt, *J*=17.9, 9.5, 4H), 7.83 – 7.70 (m, 4H), 7.63 – 7.50 (m, 4H), 7.46 – 7.27 (m, 12H), 7.20 (d, *J*=7.0, 2H), 6.97 – 6.78 (m, 6H). **2-CHO (yield: 62%):** ^{1}**H NMR** (500 MHz, $CDCl_3$) δ 10.06 (d, *J* = 10.9 Hz, 2H), 7.96 (dd, *J* = 14.4, 7.8 Hz, 4H), 7.79 – 7.73 (m, 4H), 7.64 (d, *J* = 7.7 Hz, 2H), 7.57 (s, 2H), 7.46 – 7.27 (m, 14H), 7.24 – 7.13 (m, 2H), 6.29 (d, *J* = 8.6 Hz, 2H), 3.22 (d, *J* = 4.9 Hz, 6H).

Products (1-ECN, 2-ECN)[2]**.** The product (D-CN and OD-CN) came from Knoevenagel Reaction. In detail, the compound (1-CHO or 1o-CHO (0.3 g, 0.43 mmol)) and malonitrile (0.14 g, 2.12 mmol) reacted with Et_3N (0.24ml) in CH_2Cl_2 (10ml) at room temperature for 4 h under N_2. The solvent was removed by reduced pressure evaporation. Then, the crude product was purified by chromatography using petroleum ether/ dichloromethane. The orange (D-CN) or dark-red (OD-CN) products were obtained. **1-ECN (yield: 60%):** ^{1}**H NMR** (500 MHz, $CDCl_3$) δ = 7.99 (dd, *J*=17.4, 7.6, 4H), 7.85 – 7.71 (m, 6H), 7.59 (t, *J*=9.0, 4H), 7.48 – 7.26 (m, 12H), 7.21 (s, 2H), 7.00 – 6.78 (m, 6H). ^{13}**C NMR** (126 MHz, $CDCl_3$) δ 159.13, 146.80, 146.72, 144.13, 143.12, 141.95, 141.91, 141.78, 141.46, 139.95, 137.76, 137.72, 136.04, 135.94, 135.82, 131.48, 131.42, 130.32, 129.88, 129.83, 129.35, 128.83, 128.57, 128.35, 128.30, 127.81, 127.76, 127.39, 127.07, 113.95, 112.87, 82.04, 81.90. **GCMS (EI)**: m/z calcd. $[M]^+$ 738.89, found 738.28. **2-ECN** (yield: 29%): ^{1}**H NMR** (500 MHz, $CDCl_3$) δ = 8.04 – 7.95 (m, 4H), 7.79 (d, *J*=13.0, 6H), 7.61 (dd, *J*=28.5, 7.5, 4H), 7.48 – 7.27 (m, 13H), 7.25 – 7.10 (m, 3H), 6.28 (s, 2H), 3.22 (d, *J*=5.5, 6H). ^{13}**C NMR** (126 MHz, $CDCl_3$) δ = 159.06, 159.01, 151.15, 151.09, 151.06, 146.85, 146.80, 146.76, 144.24, 144.20, 143.00, 142.13, 142.09, 141.71, 141.47, 141.42, 140.58, 137.69, 137.59, 137.56, 131.58, 131.50, 131.45, 130.48, 129.92, 129.77, 129.60, 128.90, 128.37, 128.26, 127.82, 127.75, 127.73, 127.54, 127.38, 126.98, 125.57, 123.38, 123.14, 123.04, 113.94, 113.85, 112.84, 112.79, 112.52, 112.32, 112.27, 82.19, 81.85, 55.27, 55.21, 55.14. **GCMS (EI)**: m/z calcd. $[M]^+$ 798.95, found 798.12.

Analysis on the configurations of CEOCH derivatives

After careful evaluating the multi-peaks of central methoxyl groups on 1H NMR spectra of CEOCH derivatives (2-Me, 2-OMe, 2-NMe2, 2-Ph and 2-ECN), it is inferred several stereoisomers of CEH and CEOCH exist and their substituents could be located on P1/P2 or P3/P4 (Figure S1 and Figure S2).

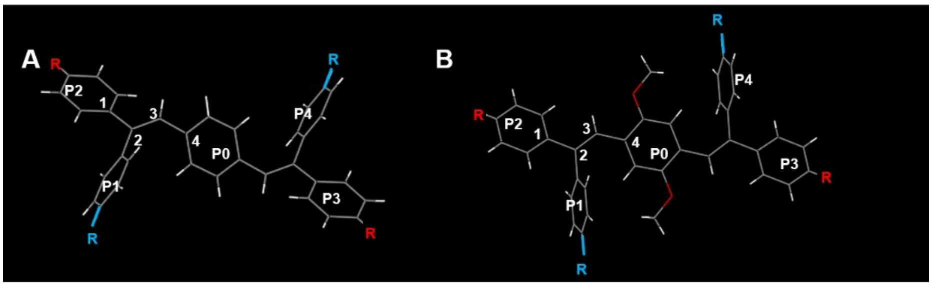

Figure S1. The structures of CEH and CEOCH derivatives.

However, through further analysis of chemical shifts of hydrogen atoms on central –OCH3 in ^{1}H NMR spectra of CEOCH derivatives in Figure S2, we found that the species which have smaller ^{1}H chemical shifts are dominated. Via comparisons the chemical shifts of central methoxyl groups on 2-H and 2-2OMe (Table S1), we have found that when central $–OCH_3$ are shielded by benzene, its ^{1}H chemical shift is 3.2 ppm; while central $–OCH_3$ are shielded by methoxybenzene, its ^{1}H chemical shift is 3.27 ppm. Thus, we think CEOCH derivatives are mainly shielded by

benzenes and functionalized on P2 and P3 phenyls.

Moreover, due to the similar chemical shifts of hydrogen on central methoxyl groups and central benzene rings of all CEOCH derivatives in different configurations, it can be inferred that no matter which benzenes (P1 - P4) of CEOCH derivatives are functionalized, the shielding effect are extremely similar and there are IMWHBs in all CEOCHs (Table S1). Then, because ^{1}H chemical shifts of central methoxyl of CEOCH derivatives are all up-field during cooling (Figure S3-S6), the hypothesis that no matter which configurations CEOCH derivatives are in, their IMWHBs are similar is also proved.

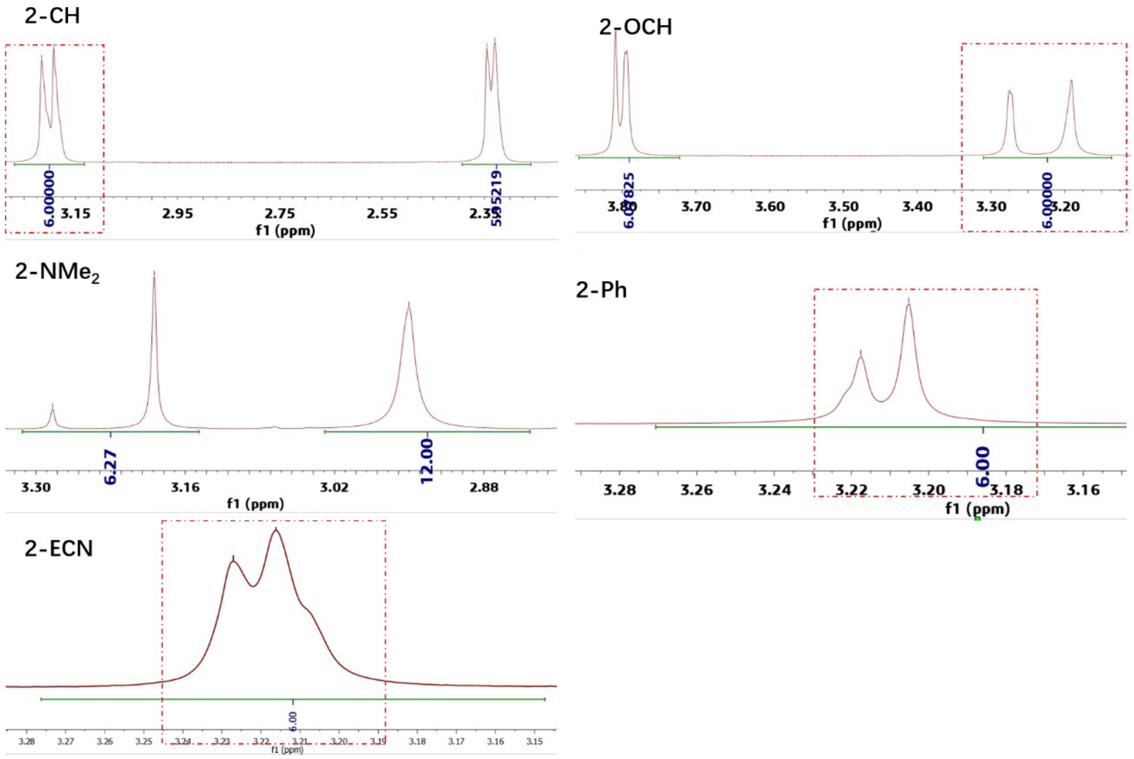

Figure S2. The enlarged ^{1}H NMR spectra of CEOCH derivatives.

Table S1. Chemical shift of hydrogen atoms on central benzene and central $–OCH_3$ of CEOCH derivatives (500 MHz, CDCl3, 25 oC).

Molecules	Chemical shift of H atoms	
	-OCH3	-Ph (central)
2-H	3.2	6.25
2-Me	3.2	6.25
2-OMe	3.19, 3.27, 3.28	6.22, 6.32
2-NMe_2	3.19, 3.28	6.20, 6.22
2-2OMe	3.27	6.30
2-Ph	3.21, 3.22	6.28, 6.31, 6.32

2-ECN	3.22, 3.23	6.28

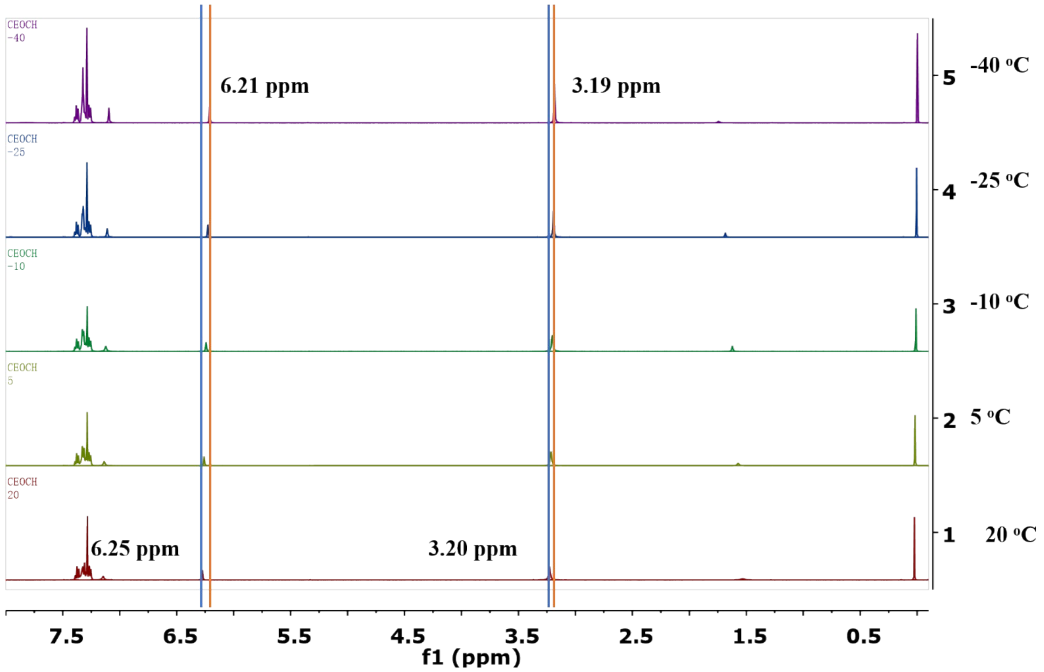

Figure S3. VT ^{1}H NMR spectra of 2-H in $CDCl_3$.

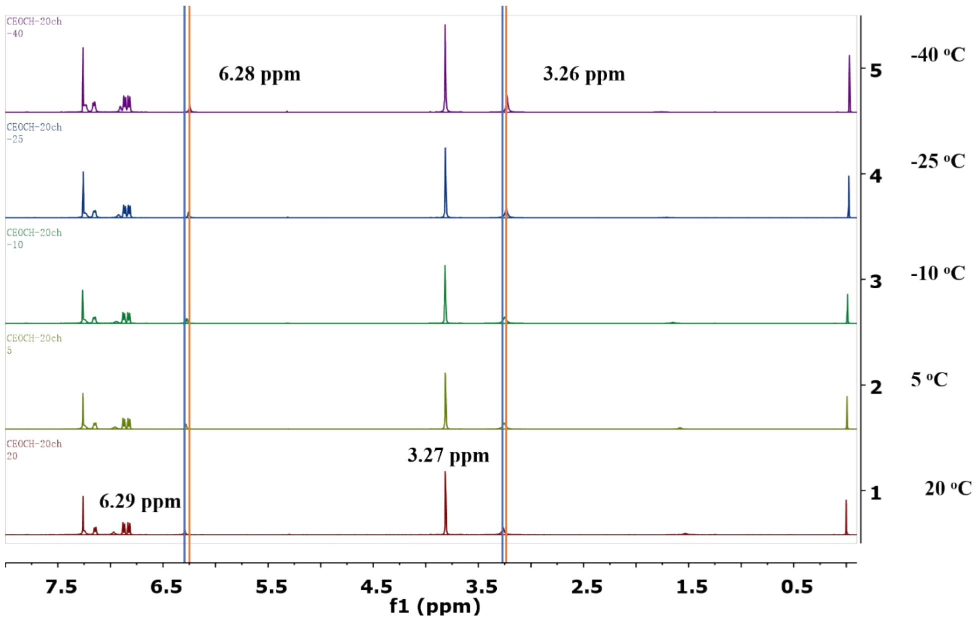

Figure S4. VT ^{1}H NMR spectra of 2-2OMe in $CDCl_3$.

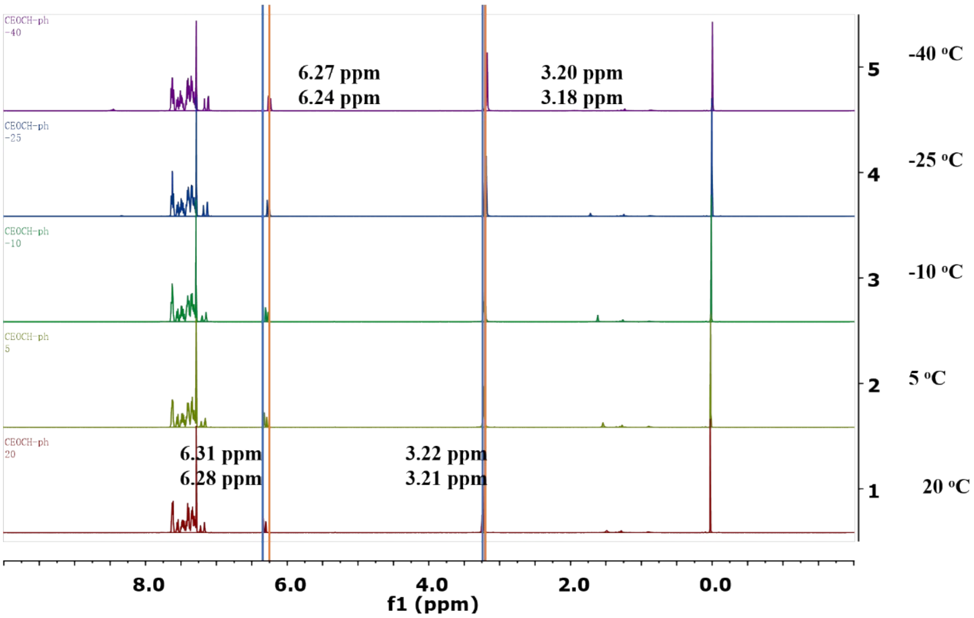

Figure S5. VT ^{1}H NMR spectra of 2-Ph in $CDCl_3$.

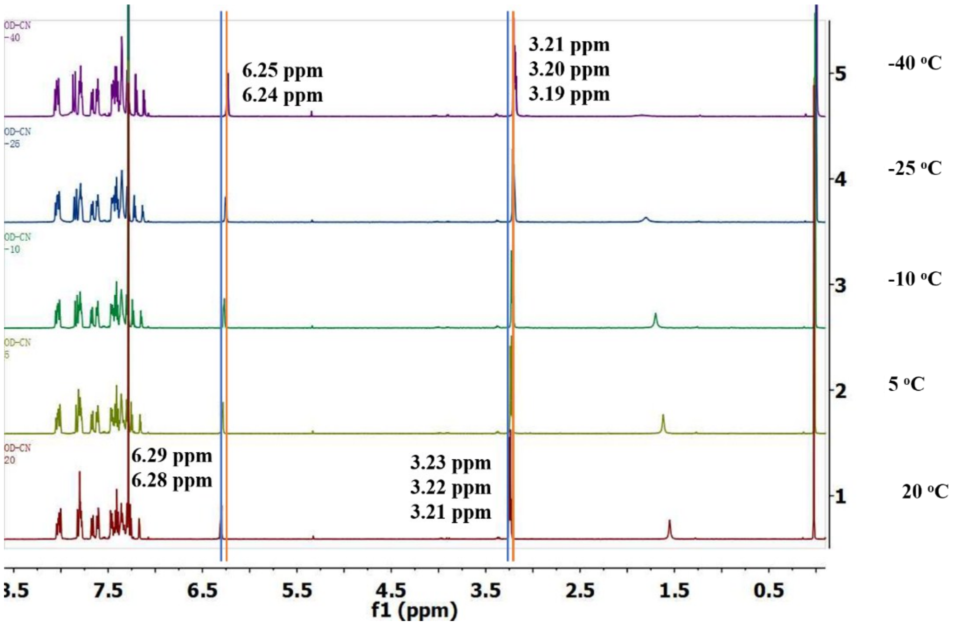

Figure S6. VT ^1H NMR spectra of 2-ECN in $CDCl_3$.

Further, we have conducted the calculations[3] of substituted CEOCH on different configurations and found the energy of 2-ECN with extended P2 and P3 phenyls is smallest (Figure S7), which also suggest the configuration of substituted CEOCH with extended P2 and P3 are more favorable.

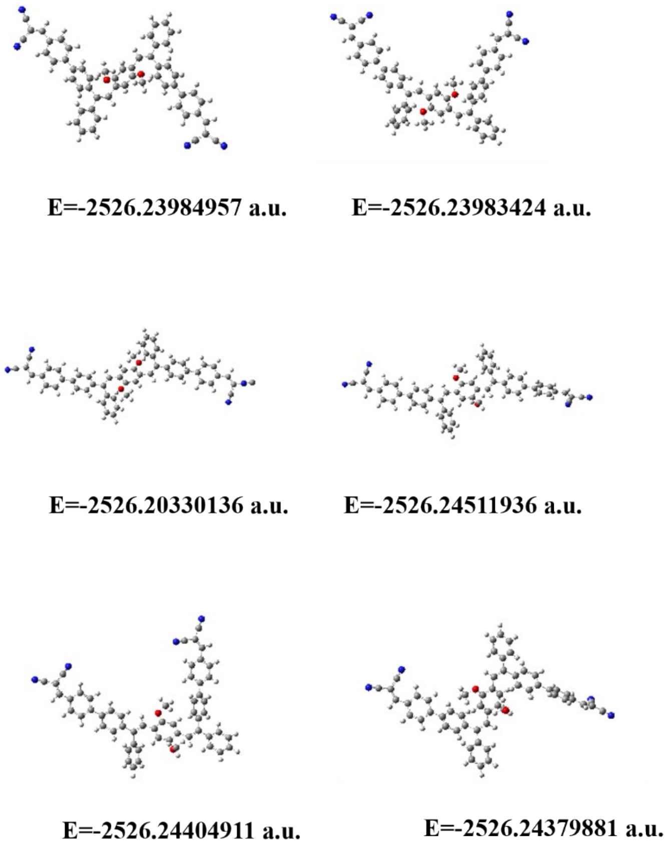

Figure S7. The isomers and their energies of 2-ECN.[3]

Moreover, we are very lucky to obtain the single crystal structure of 2-NMe_2 (CCDC number: 1906459) and find 2-NMe_2 has a large preference toward one configuration as disclosed in NMR spectra. Therefore, the structure of 2-NMe_2 is as illustrated in Scheme 1 and functionalized on P2 and P3 phenyls.

Thus, by comprehensive consideration of NMR spectra, calculation and single crystal data, the configurations of CEOCH derivatives with extended P2 and P3 phenyls are more favorable. In addition, owing to the similar behavior of ^1H VT-NMR of CEOCH derivatives, the extended P2 and P3 phenyls of CEH and CEOCH derivatives could be used in the following calculations related to IMWHBs.

After calculated the optimized structures of CEH and CEOCH derivatives, the twisted angles between P0 and bordering benzenes are in the region of 60° -70°, proving the structures of CEH and CEOCH derivatives are twisted (Figure S1).

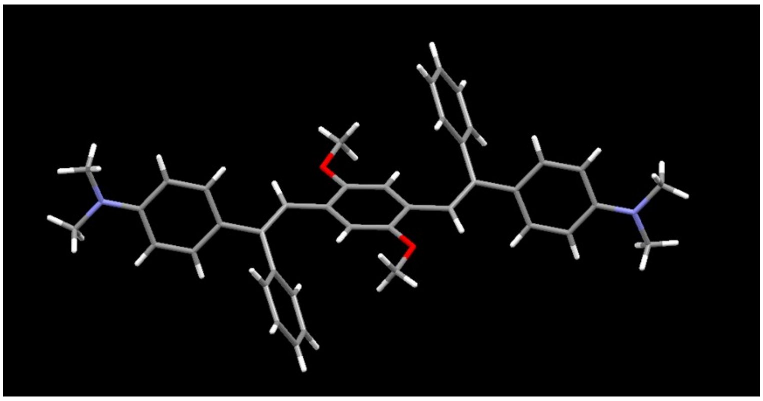

Figure S8. The crystal structures of 2-NMe_2.

The photophysical properties of CEOCH and CEH derivatives

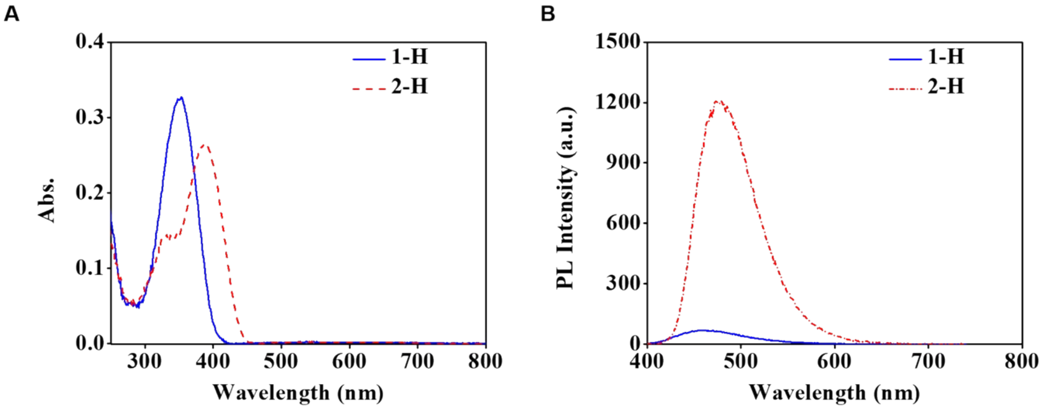

Figure S9. The absorption (A) and emission (B) spectra of 1-H and 2-H in THF solution (10 uM). Excitation wavelength: 380nm. Slits: 3, 3.

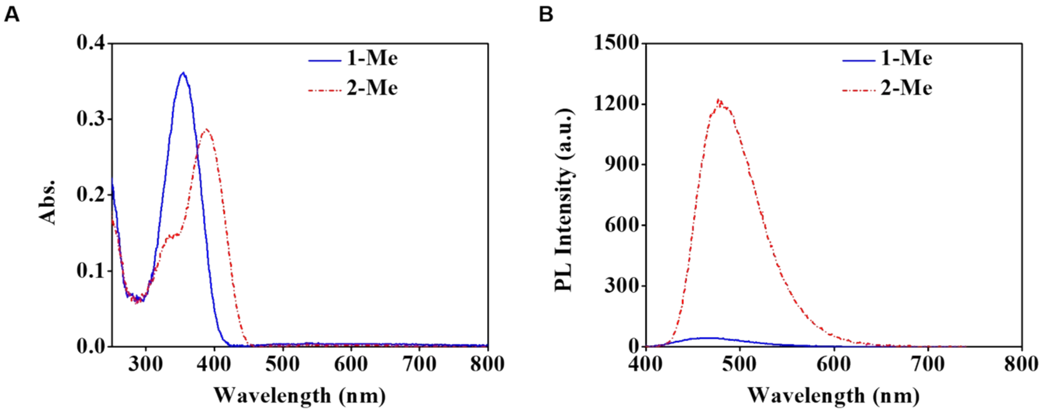

Figure S10. The absorption (A) and emission (B) spectra of 1-Me and 2-Me in THF solution (10 uM). Excitation wavelength: 380nm. Slits: 3, 3.

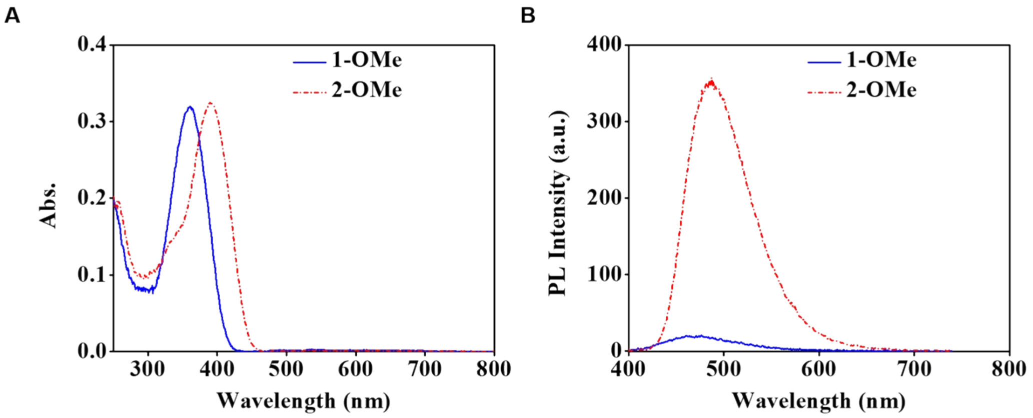

Figure S11. The absorption (A) and emission (B) spectra of 1-OMe and 2-OMe in THF solution (10 uM). Excitation wavelength: 380nm. Slits: 3, 3.

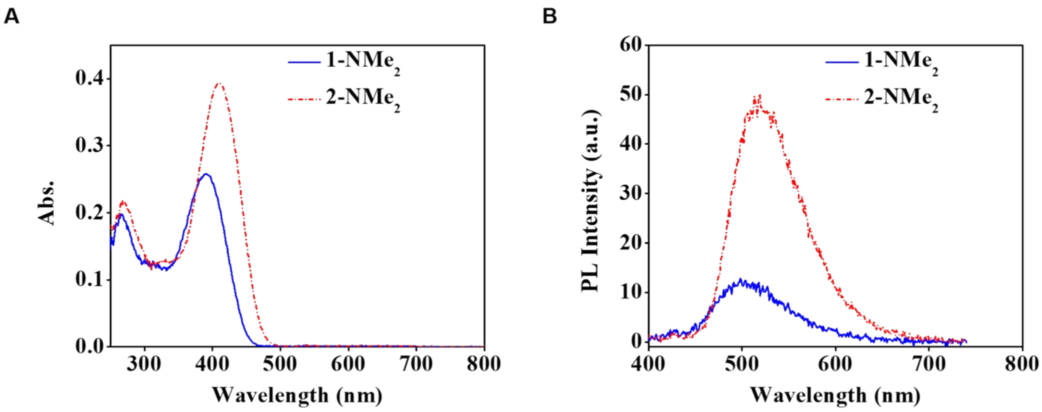

Figure S12. The absorption (A) and emission (B) spectra of 1-NMe_2 and 2-NMe_2 in THF solution (10 uM). Excitation wavelength: 380nm. Slits: 3, 3.

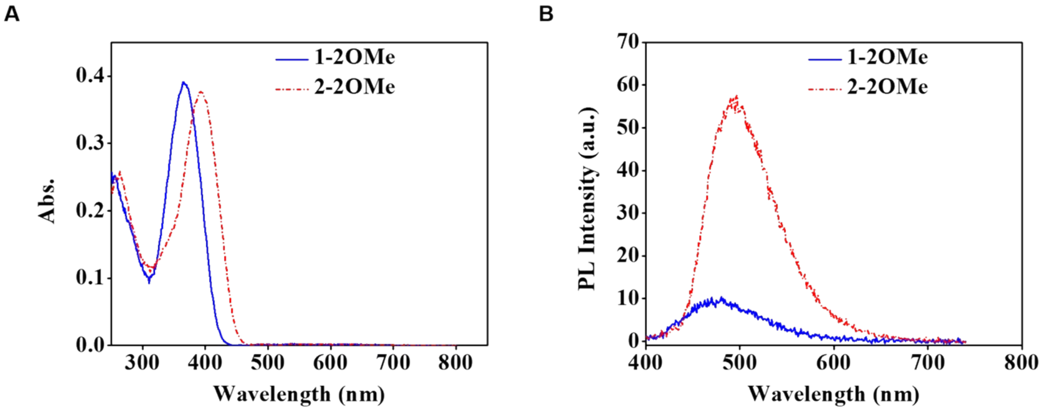

Figure S13. The absorption (A) and emission (B) spectra of 1-2OMe and 2-2OMe in THF solution (10 uM). Excitation wavelength: 380nm. Slits: 3, 3.

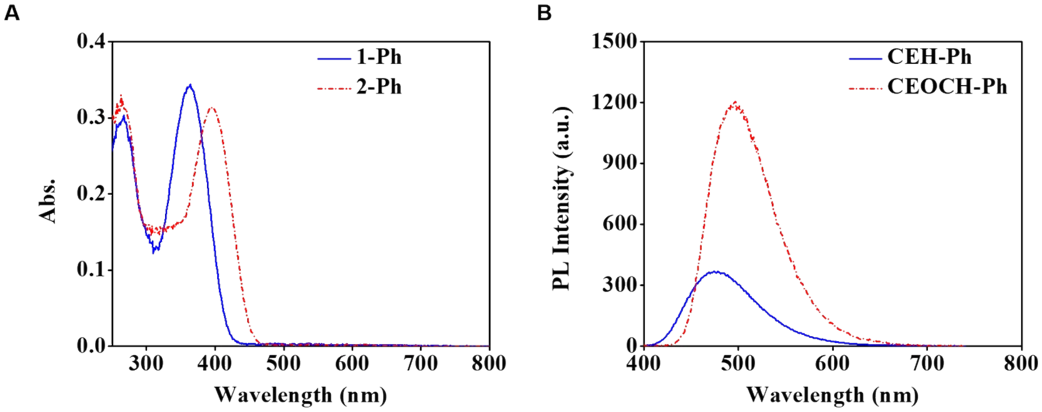

Figure S14. The absorption (A) and emission (B) spectra of 1-Ph and 2-Ph in THF solution (10 uM). Excitation wavelength: 380nm. Slits: 3, 3.

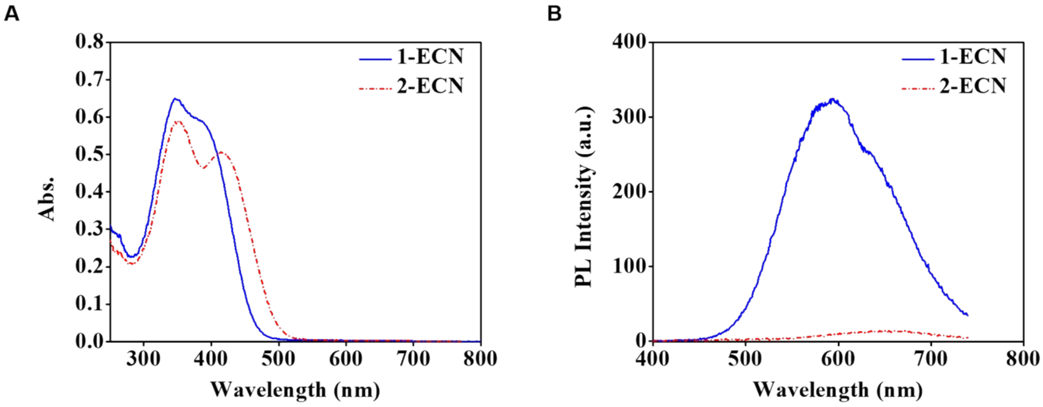

Figure S15. The absorption (A) and emission (B) spectra of 1-ECN and 2-ECN in THF solution (10 uM). Excitation wavelength: 380nm. Slits: 3, 3.

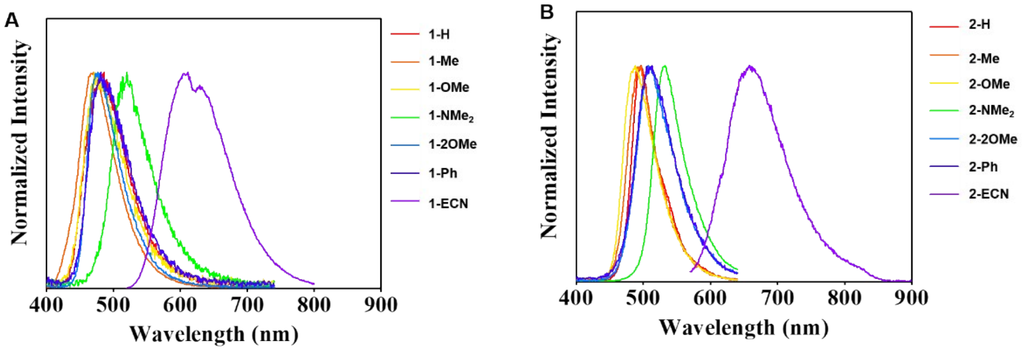

Figure S16. The emission spectra of CEH derivatives (A) and CEOCH derivatives (B) in solid state. Excitation wavelength: 380 nm for all molecules except for D-CN (500 nm) and OD-CN (550 nm).

Calculations

The optimization of ground states and excited states of CEH and CEOCH derivatives were calculated based on B3LYP/6-31G(d,p) level, Gaussian 09 Program.[3] On the optimized geometry of the ground state and the excited state, the single point energies[3] of CEH and CEOCH derivatives were carried out with B3LYP/6-31G(d,p) level, Gaussian 09 Program, too.

Table S2. Calculated absorption and emission data for CEH and CEOCH derivatives based on B3LYP/6-31G(d,p) level, Gaussian 09 Program.[3]

	Absorption		Emission	
	Electronic state transition	Vertical excitation energy (nm)	Electronic state transition	Vertical excitation energy (nm)
**1-H**	$S_0 \rightarrow S_1$	391	$S_1 \rightarrow S_0$	524
**2-H**	$S_0 \rightarrow S_1$	421	$S_1 \rightarrow S_0$	539
**1-Me**	$S_0 \rightarrow S_1$	394.6	$S_1 \rightarrow S_0$	529.26
**2-Me**	$S_0 \rightarrow S_1$	424.99	$S_1 \rightarrow S_0$	545.1
**1-OMe**	$S_0 \rightarrow S_1$	404.17	$S_1 \rightarrow S_0$	535.1
**2-OMe**	$S_0 \rightarrow S_1$	428.7	$S_1 \rightarrow S_0$	552.21
**1-NMe2**	$S_0 \rightarrow S_1$	431.3	$S_1 \rightarrow S_0$	553.13
**2-NMe2**	$S_0 \rightarrow S_1$	384	$S_1 \rightarrow S_0$	573.95
**1-2OMe**	$S_0 \rightarrow S_1$	410.73	$S_1 \rightarrow S_0$	537.23
**2-2OMe**	$S_0 \rightarrow S_1$	430.98	$S_1 \rightarrow S_0$	563.17
**1-Ph**	$S_0 \rightarrow S_1$	413.51	$S_1 \rightarrow S_0$	546.52
**2- Ph**	$S_0 \rightarrow S_1$	440.28	$S_1 \rightarrow S_0$	566.39
**1-ECN**	$S_0 \rightarrow S_1$	535.22	$S_1 \rightarrow S_0$	634.69
**2-ECN**	$S_0 \rightarrow S_1$	563.58	$S_1 \rightarrow S_0$	673.16

The calculated absorption and emission peaks of these molecules by B3LYP/6-31G (d,p) level (Table S6) fit well with the experimental data, which proves the reliability of calculated results.

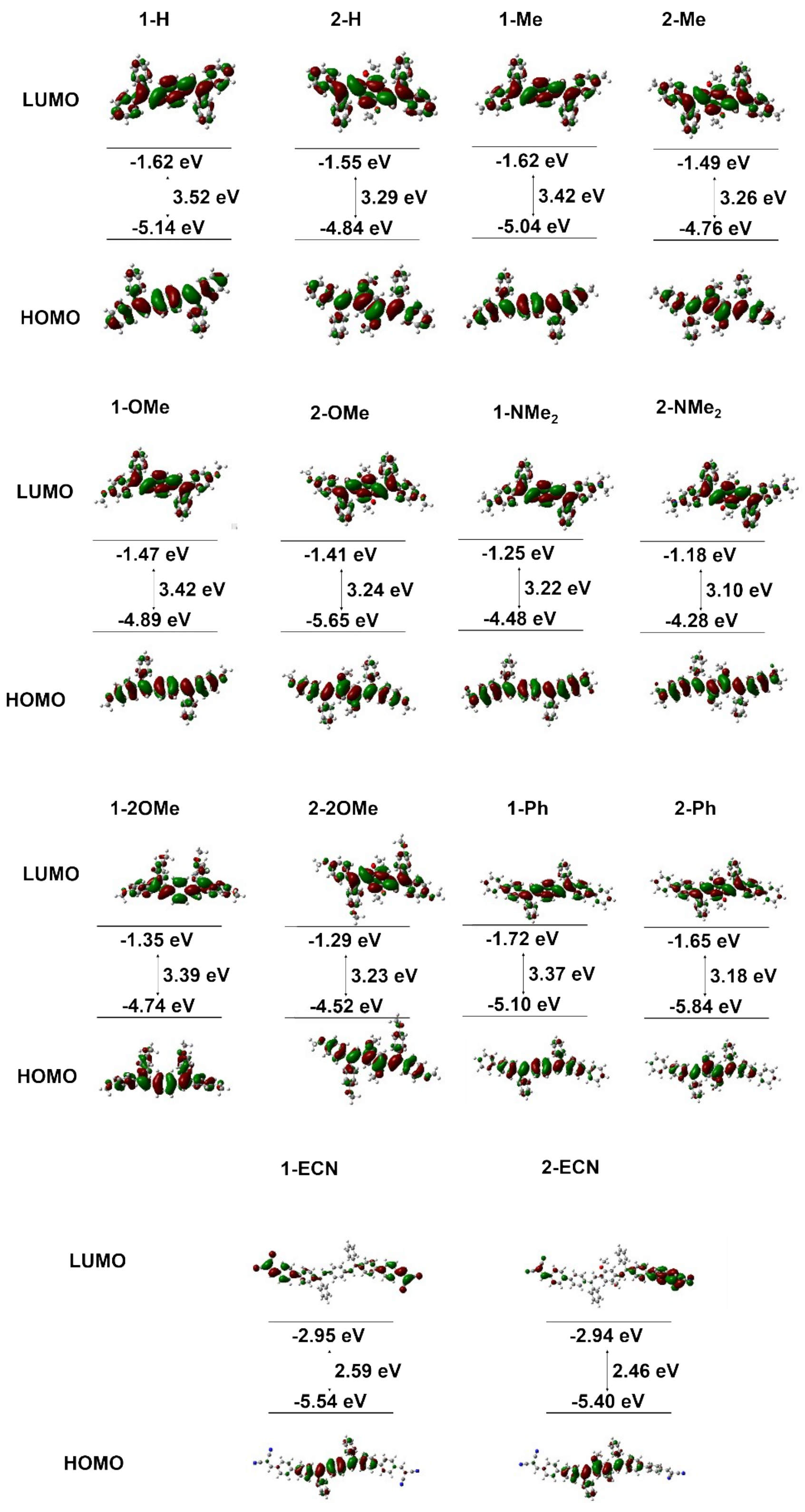

Figure S17. Calculated molecular orbitals of the HOMO and the LUMO of CEH and CEOCH derivatives.[3]

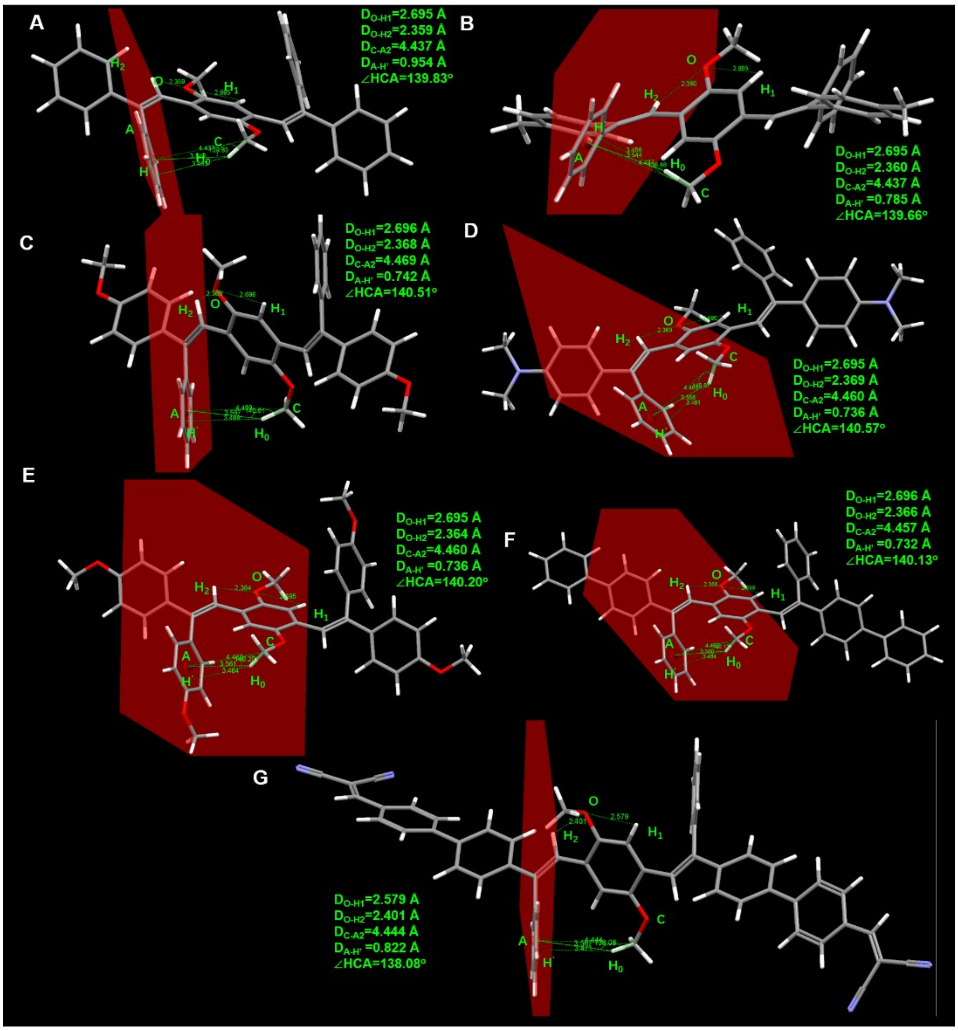

Figure S18. The intramolecular forces for 2-H (A), 2-Me(B), 2-OMe (C), 2-2NMe$_2$ (D), 2-2OMe (E), 2-Ph (F) and 2-ECN(G) in optimized ground state. O represents for the oxygen atom on central methoxyl groups; C represents for the carbon atom on central methoxyl groups; A represents for the centroid of the benzene ring; H0, H1, H2 represent for the hydrogen atoms on central methoxyl groups, central benzenes and C=C bonds; H' represents for the vertical projection of H0 on the plane of benzene ring.

In Figure S18, it can't only be seen that CEOCH derivatives are highly twisted, but there are intramolecular interactions in these molecules because the distances and angles between central -OCH3 and neighbouring groups (C=C bonds, central benzenes and bordering benzenes) are in the region of the CH…O and CH…pi interactions (CH…O interactions: distances of CH…O are less than 4 Å; CH…pi interactions: distances of C and centroid of benzenes are less than 4.5 Å, the angles of C-H-centroid of benzenes are about to 120° and the distances of the vertical projection of H on the plane of benzene ring and centroid of benzenes are less than 1.2 Å).[4]

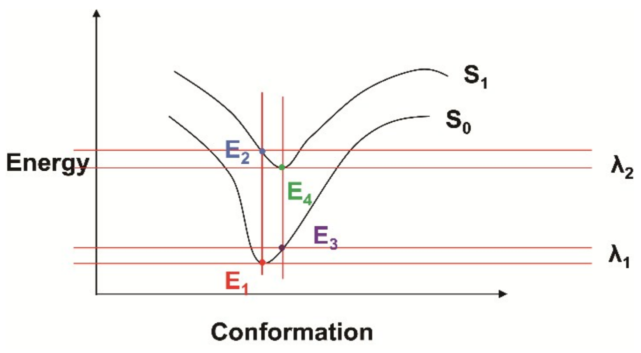

Scheme S2. Schematic illustration of adiabatic excitation energy (E4-E1) and reorganization energy ($\lambda=\lambda1+\lambda2$; $\lambda1$=E3-E1, representing the contribution of the ground state to the total reorganization energy; $\lambda2$=E2-E4, representing the contribution of the excited state. E1 represents the minimum energy of ground state (S0), E2 represents the energy of excited state at S0 minimum conformation, E3 represents for the energy of ground state at S1 minimum conformation and E4 represents for the minimum energy of excited state (S1).[1,5-8]

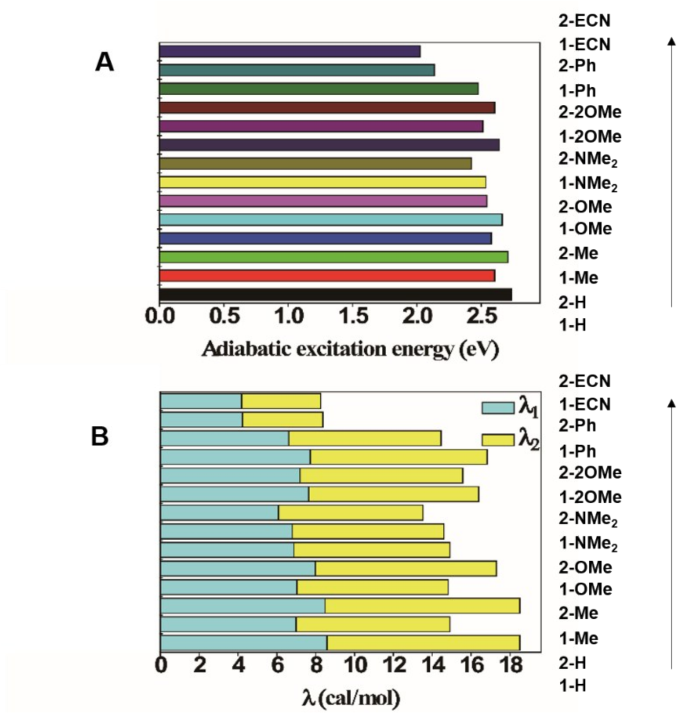

Figure S19. The adiabatic excitation energy (A) and reorganization energy, λ (B) of isolated CEH and CEOCH derivatives. $\lambda1$ and $\lambda2$ represent for the contribution of the ground state and excited state to the total reorganization energy, respectively.

Hydrazine detection

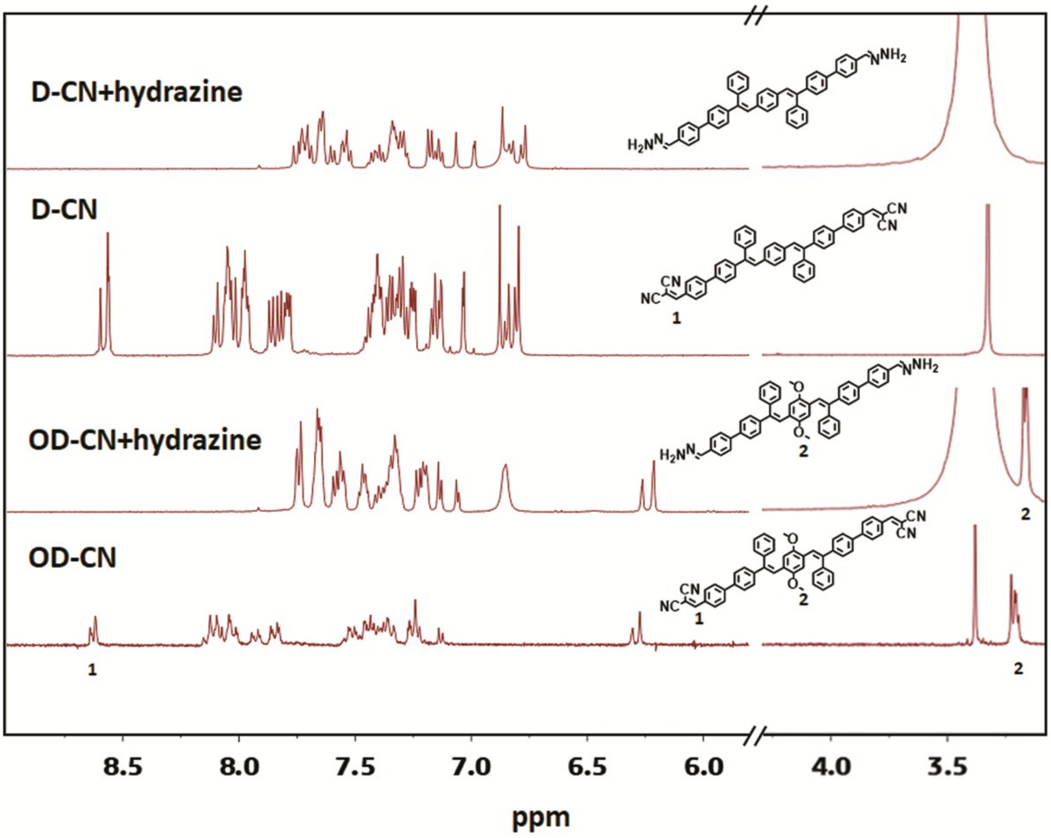

Figure S20. ^1H NMR spectra of 1-ECN and 2-ECN upon the addition of hydrazine hydrate in DMSO-d6.

That the signal of proton (>8.5 ppm) on di-cyano-vinyl groups disappeared proves the displacement reactions between 1-ECN/2-ECN and hydrazine which convert the di-cyano-vinyl groups to hydrazone groups happen.

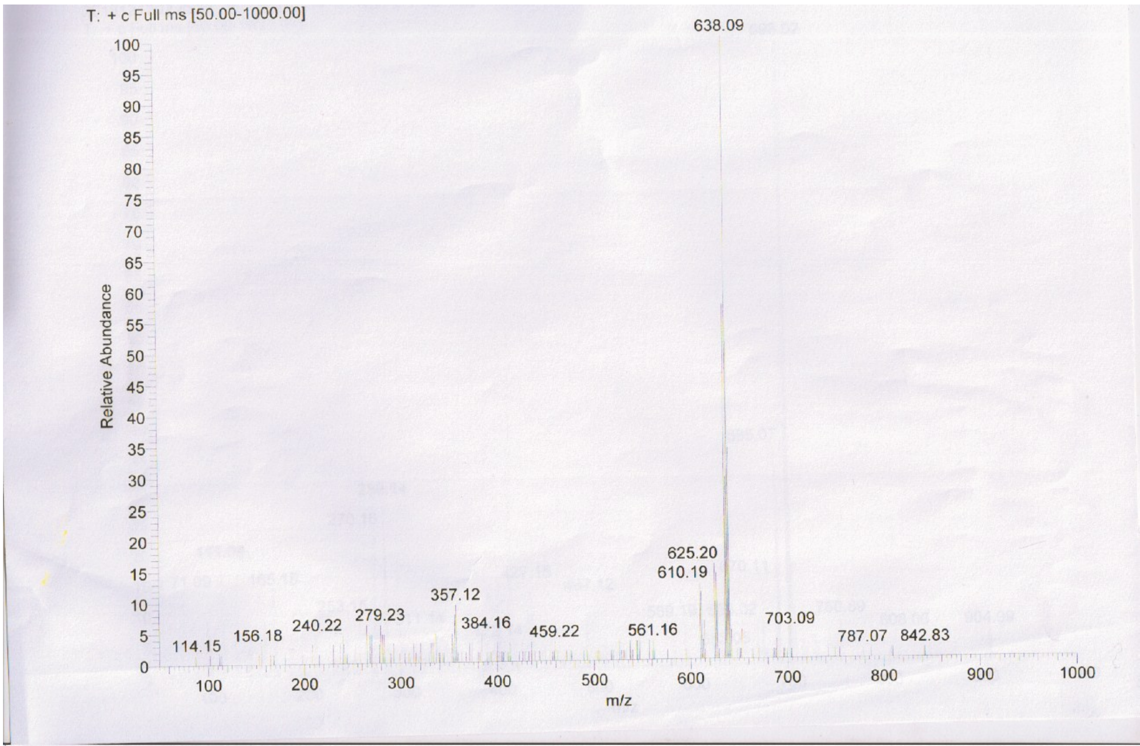

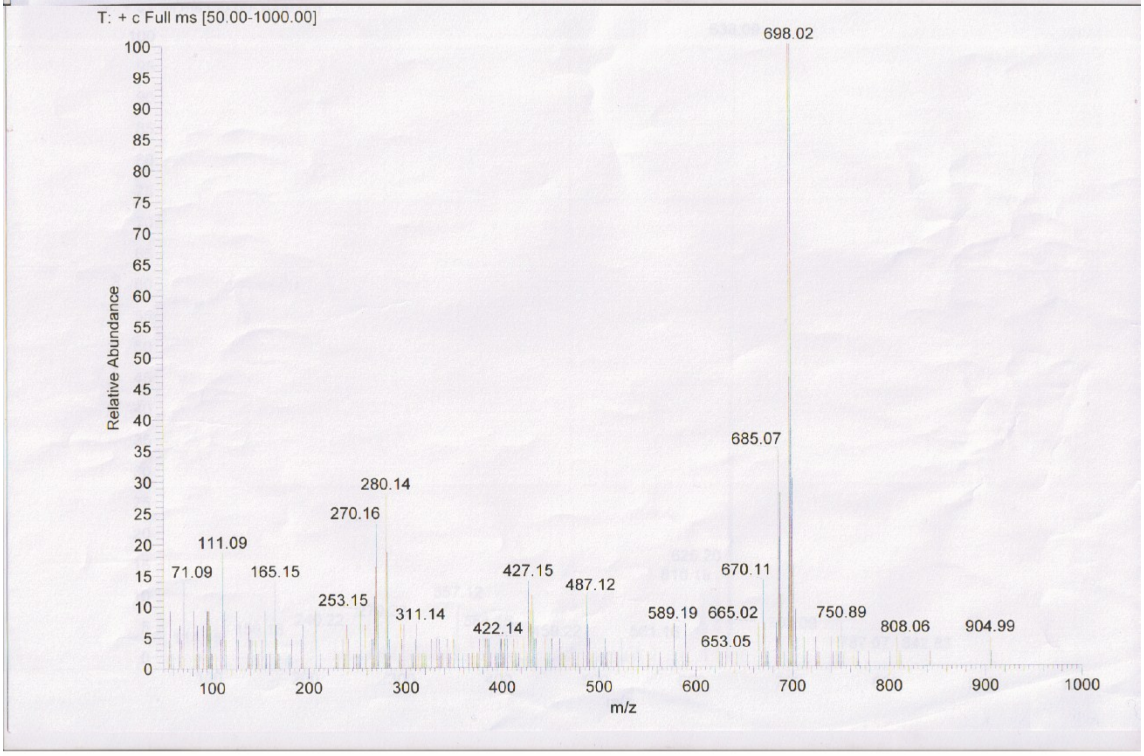

Figure S21. GCMS (EI) spectra of 1-ECN (top) and 2-ECN (bottom) upon addition hydrazine.

The new m/z peaks at 638.09 and 698.02 could be assigned to the fragment ions of hydrazine adducted 1-ECN and 2-ECN because the N-N single bonds of hydrazine adducted 1-ECN and 2-ECN are easy to be broken.

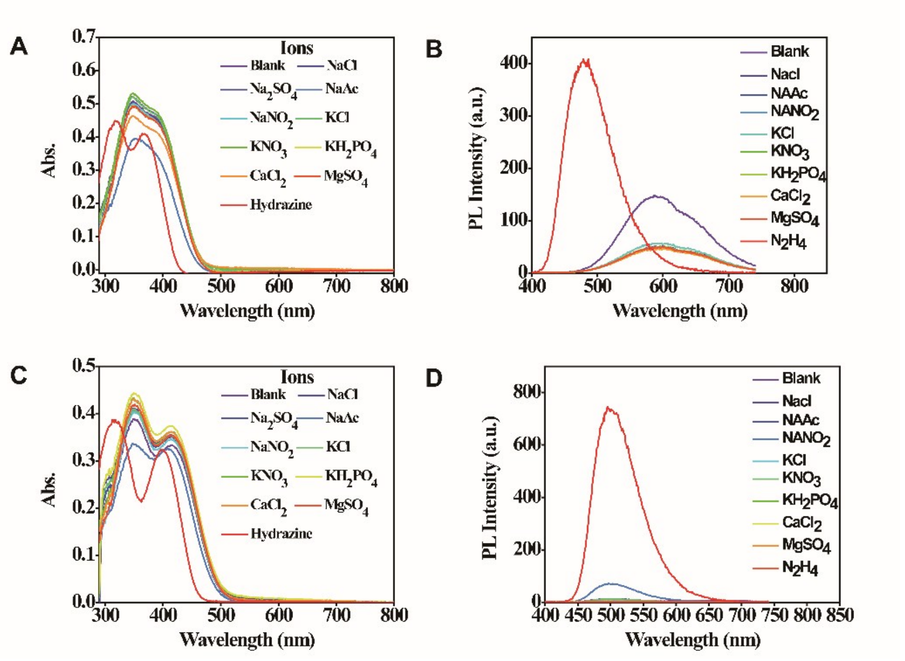

Figure S22. Absorption and emission spectra of 1-ECN (A and B) and 2-ECN (C and D) in THF solution with various ions (10 equiv.) and hydrazine (4 equiv.). Excitation wavelength: 380 nm. Slits: 1.5, 3.

In FigureS22, through analysis of absorbance at 380 nm and fluorescence-peak intensity of 2-ECN before or after adding hydrazine, the rough enhancement of QYs can be obtained (about 100 times) via the following formula.

$$T=QY_A/QY_B=QY_B(Abs_B/Abs_A)(I_A/I_B)/QY_B$$

T represents for the times of emissive enhancement after adding hydrazine; QY_A represents for the quantum yield of 2-ECN after adding hydrazine; QY_b represents for the quantum yield of 2-ECN before adding hydrazine; Abs_A represents for the absorbance of 2-ECN after adding hydrazine at 380 nm; Abs_b represents for the absorbance of 2-ECN before adding hydrazine at 380 nm; I_A represents for the intensity of the emissive peak of 2-ECN after adding hydrazine; I_B represents for the intensity of the emissive peak of 2-ECN before adding hydrazine.

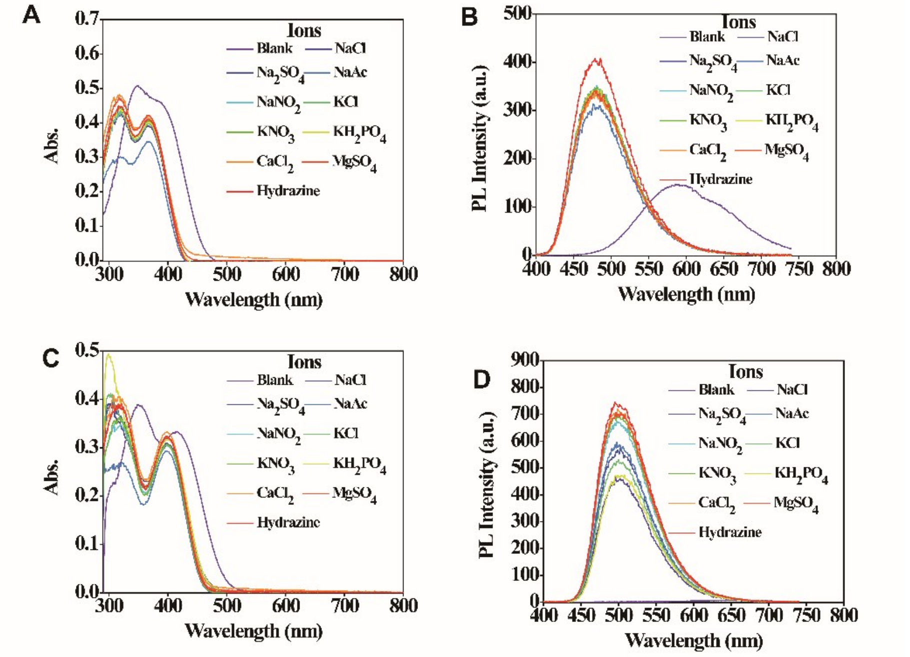

Figure S23. Absorption and emission spectra of 1-ECN (A and B) and 2-ECN (C and D) in THF solution with various ions (10 equiv.) upon addition of hydrazine (4 equiv.). Excitation wavelength: 380 nm. Slits: 1.5, 3.

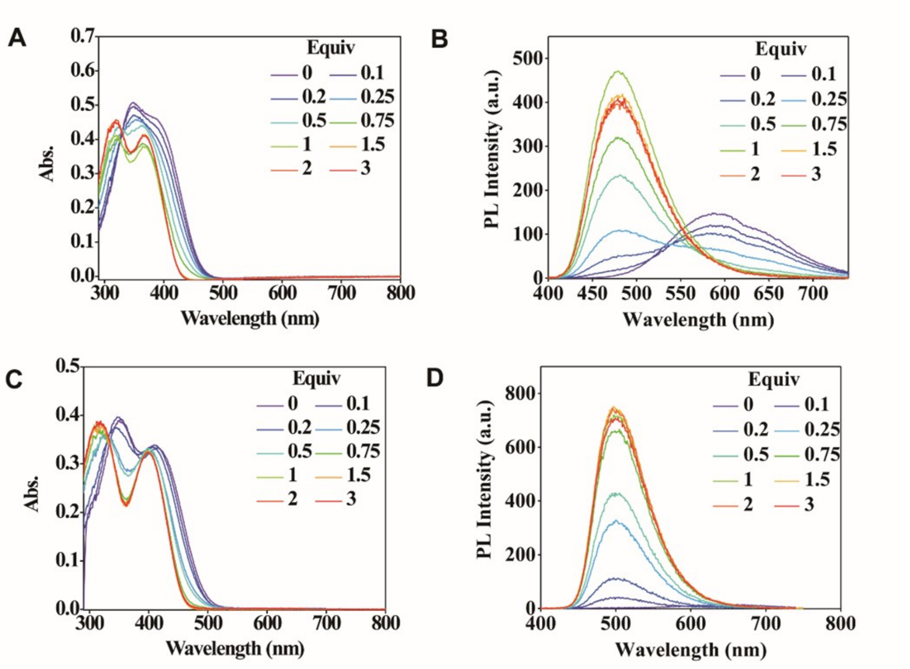

Figure S24. Absorption and emission spectra of 1-ECN (A and B) and 2-ECN (C and D) in THF solution upon addition of hydrazine with different equivalent. Excitation wavelength: 380 nm. Slits: 1.5, 3.

In Figure S22-S24, though 1-ECN and 2-ECN both show selectivity and sensitivity towards hydrazine, 2-ECN possess higher luminance contrast for the central methoxyl groups take a great part in rigidifying molecular structures, rather than free motions. Furthermore, the slight enhancement of 2-ECN upon addition of Ac^- is caused by that the nucleophilic addition reaction between basic groups and edging C=C bonds also break the electron-withdrawing properties of substituents on

bordering benzenes.

NMR spectra

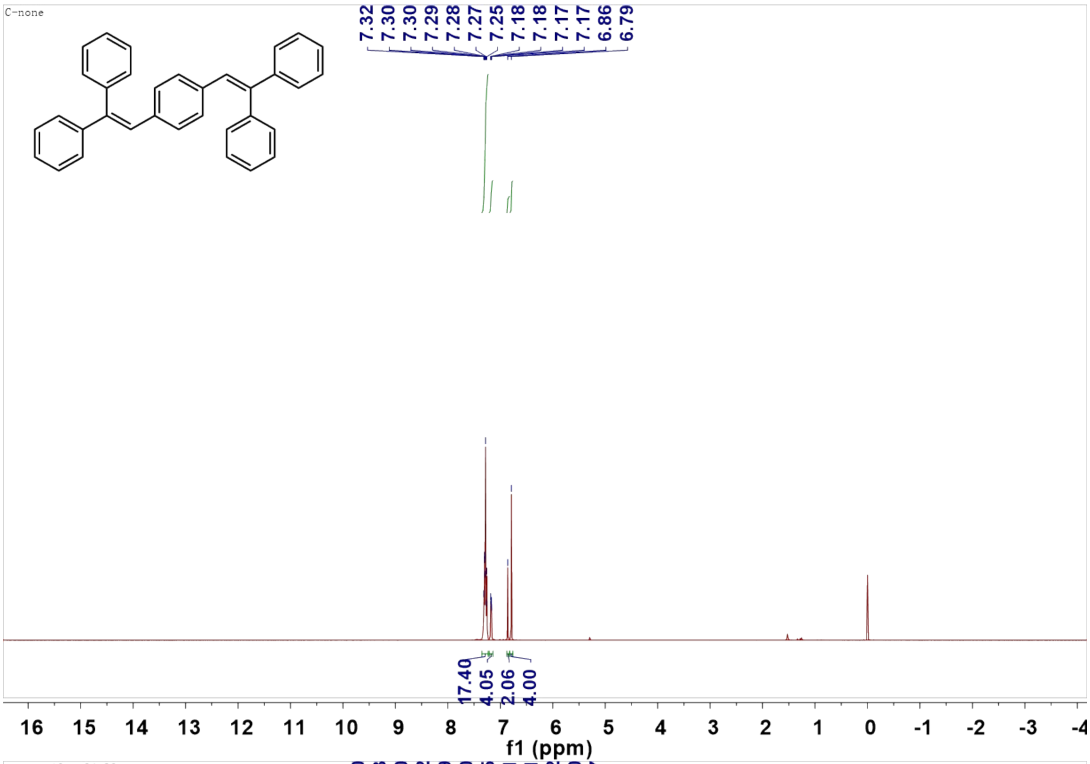

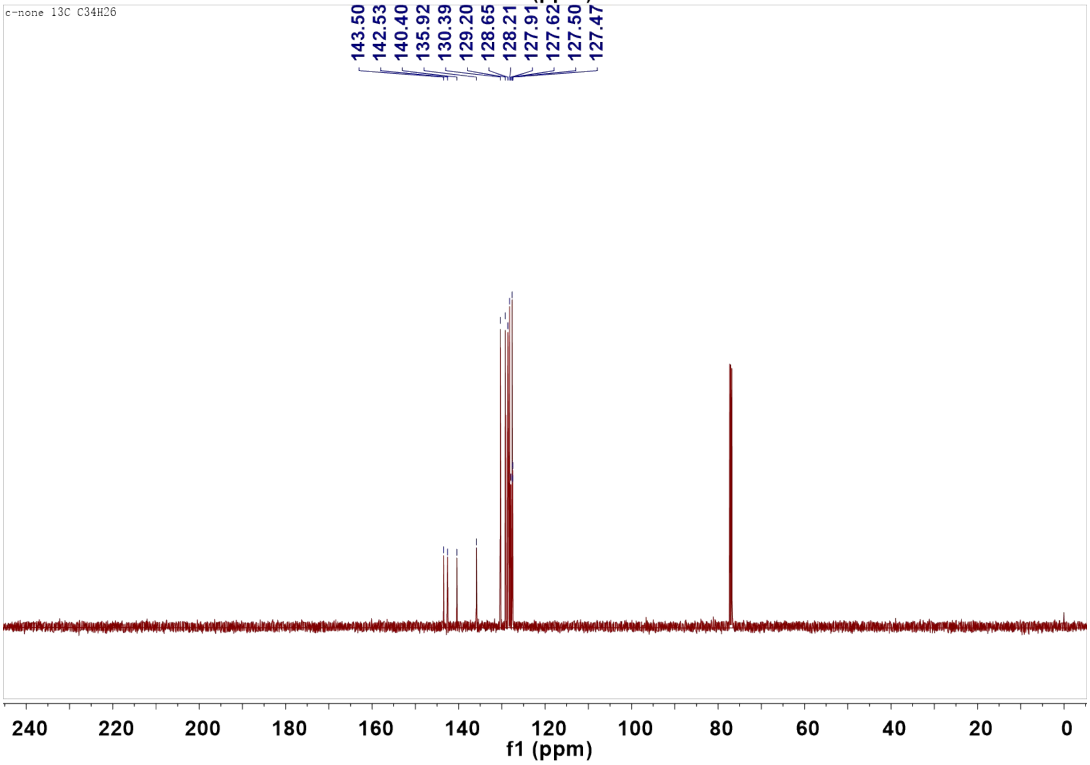

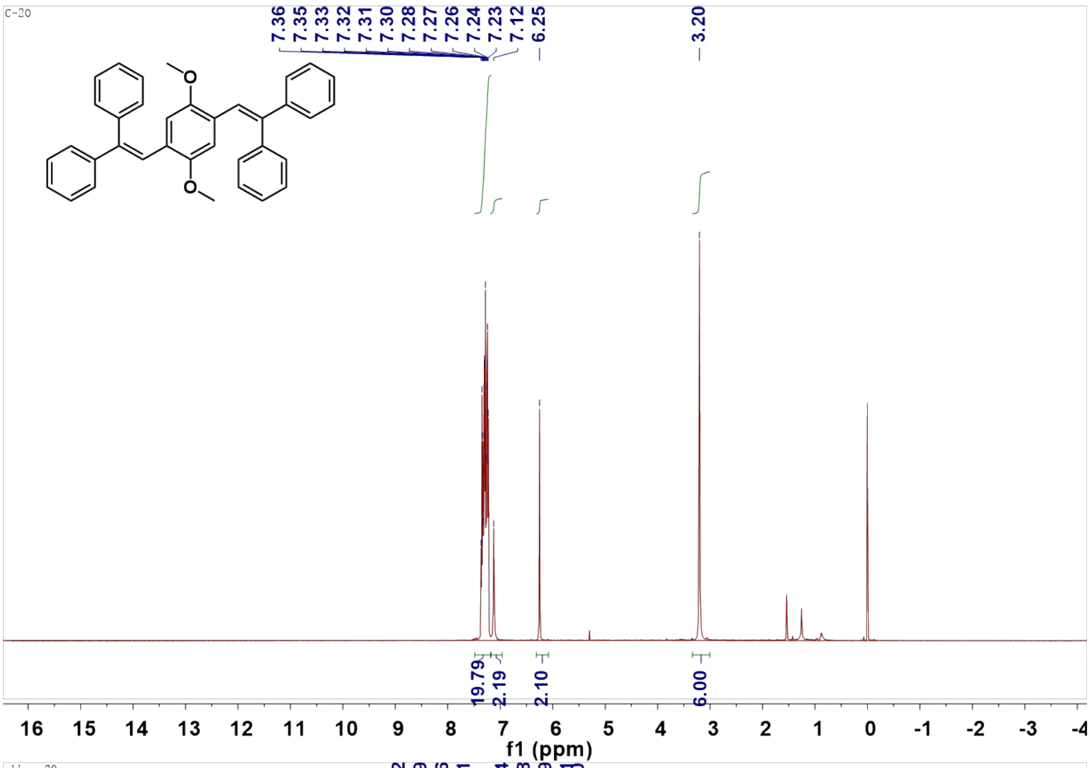
C-20
7.36
7.35
7.33
7.32
7.31
7.30
7.28
7.27
7.26
7.24
7.23
7.12
6.25
3.20
O
O
19.79
2.19
2.10
6.00
16 15 14 13 12 11 10 9 8 7 6 5 4 3 2 1 0 -1 -2 -3 -4
f1 (ppm)

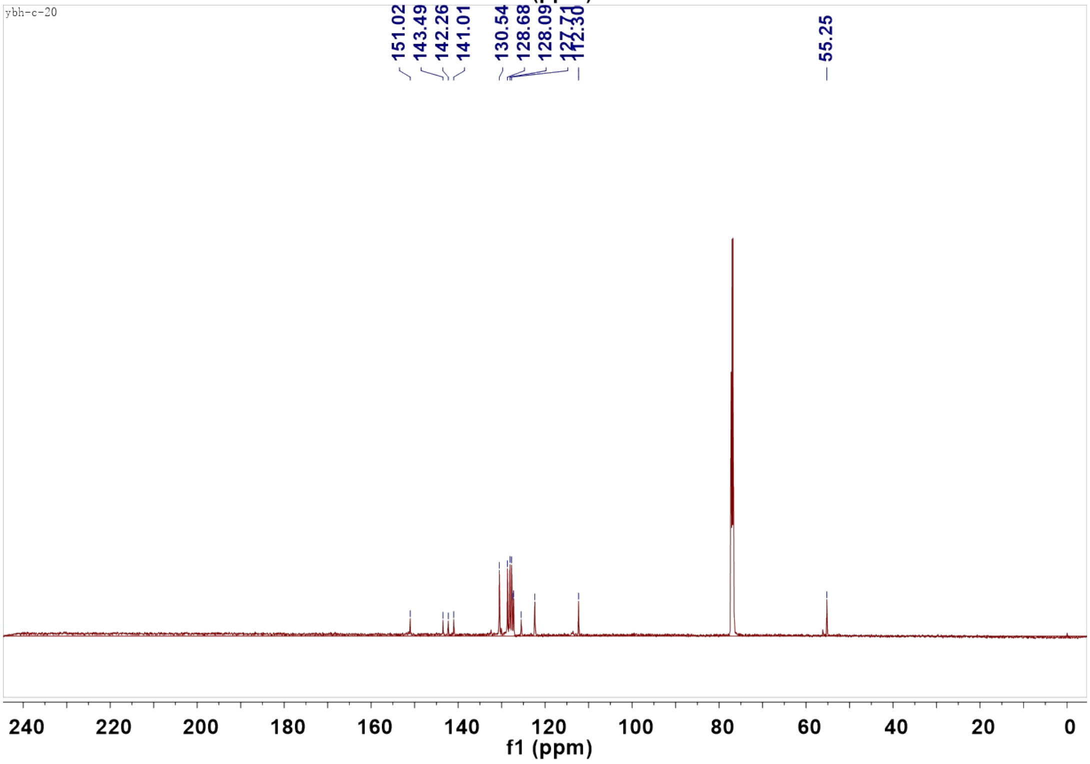
ybh-c-20
151.02
143.49
142.26
141.01
130.54
128.68
128.09
127.71
112.30
55.25
240 220 200 180 160 140 120 100 80 60 40 20 0
f1 (ppm)

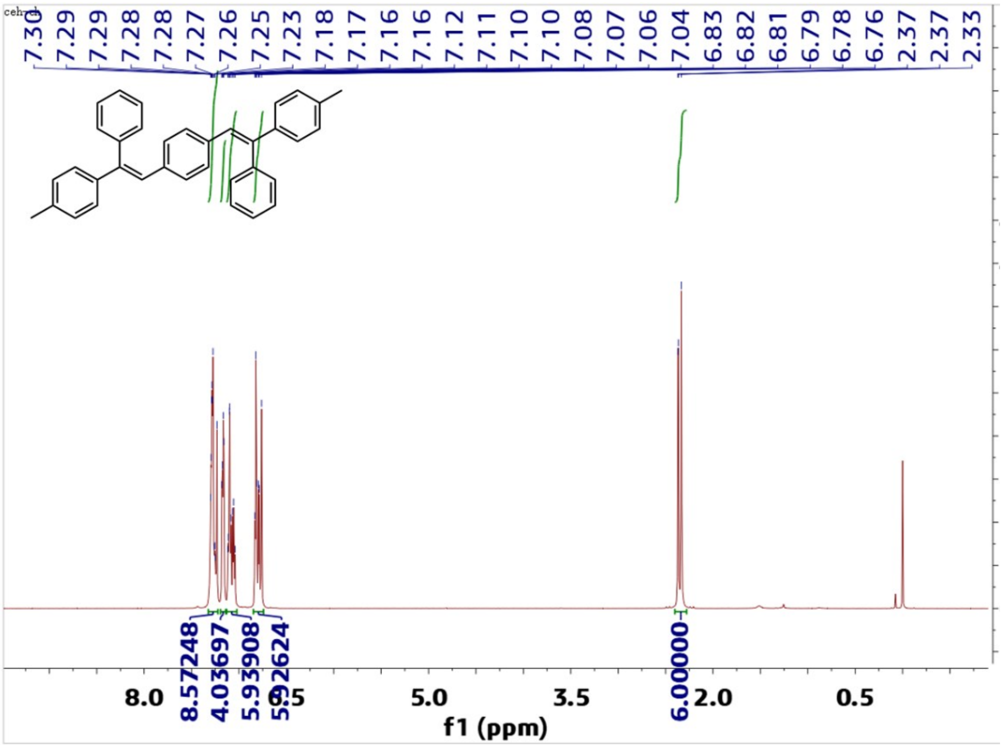
7.30
7.29
7.29
7.28
7.28
7.27
7.26
7.25
7.23
7.18
7.17
7.16
7.16
7.12
7.11
7.10
7.10
7.08
7.07
7.06
7.04
6.83
6.82
6.81
6.79
6.78
6.76
2.37
2.37
2.33
8.57248
4.03697
5.93908
5.92624
6.00000
8.0
5.0
3.5
2.0
0.5
f1 (ppm)

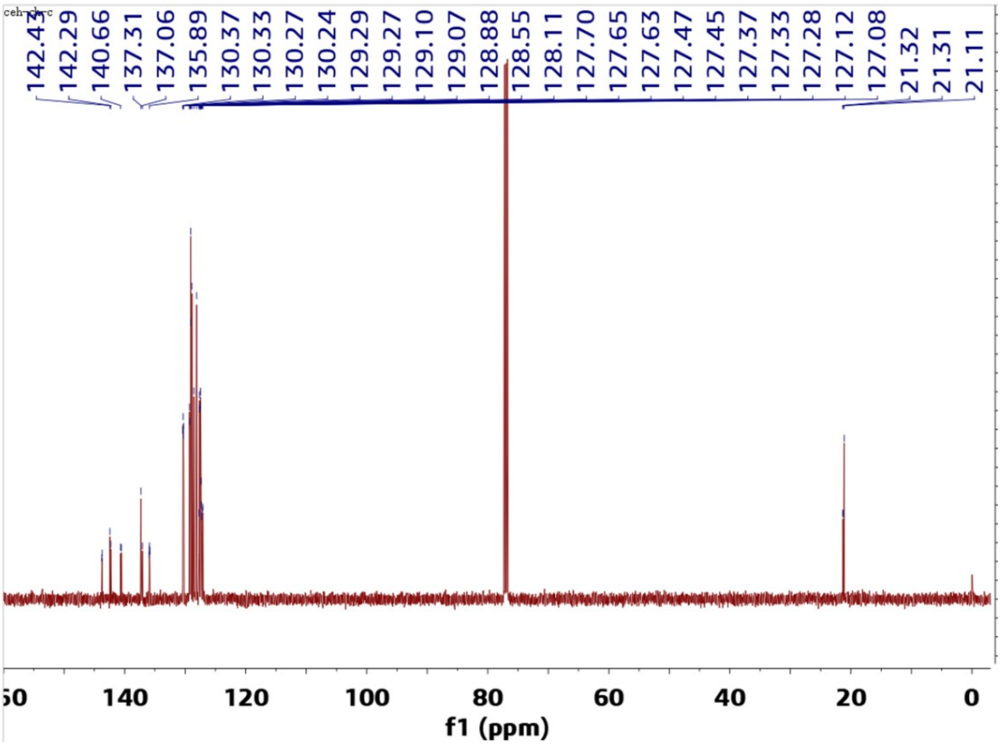
142.4
142.29
140.66
137.31
137.06
135.89
130.37
130.33
130.27
130.24
129.29
129.27
129.10
129.07
128.88
128.55
128.11
127.70
127.65
127.63
127.47
127.45
127.37
127.33
127.28
127.12
127.08
21.32
21.31
21.11
140
120
100
80
60
40
20
0
f1 (ppm)

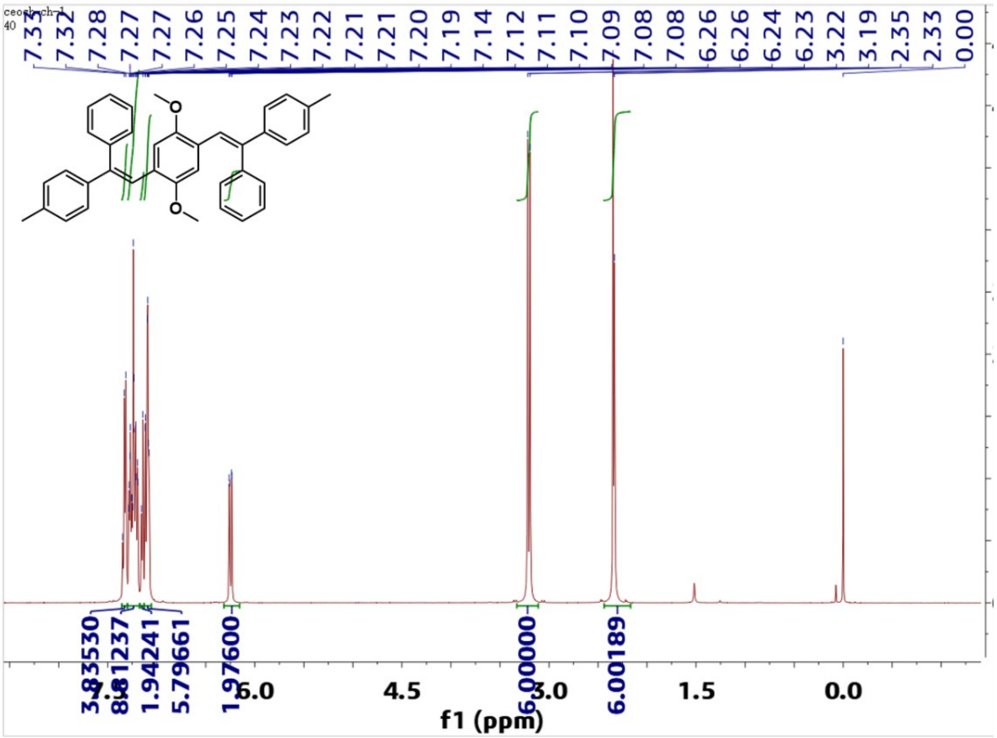

7.33
7.32
7.28
7.27
7.27
7.26
7.25
7.24
7.23
7.22
7.21
7.21
7.20
7.19
7.14
7.12
7.11
7.10
7.09
7.08
7.08
6.26
6.26
6.24
6.23
3.22
3.19
2.35
2.33
0.00
3.83530
8.81237
1.94241
5.79661
1.97600
6.00000
6.00189
7.5
6.0
4.5
3.0
1.5
0.0
f1 (ppm)

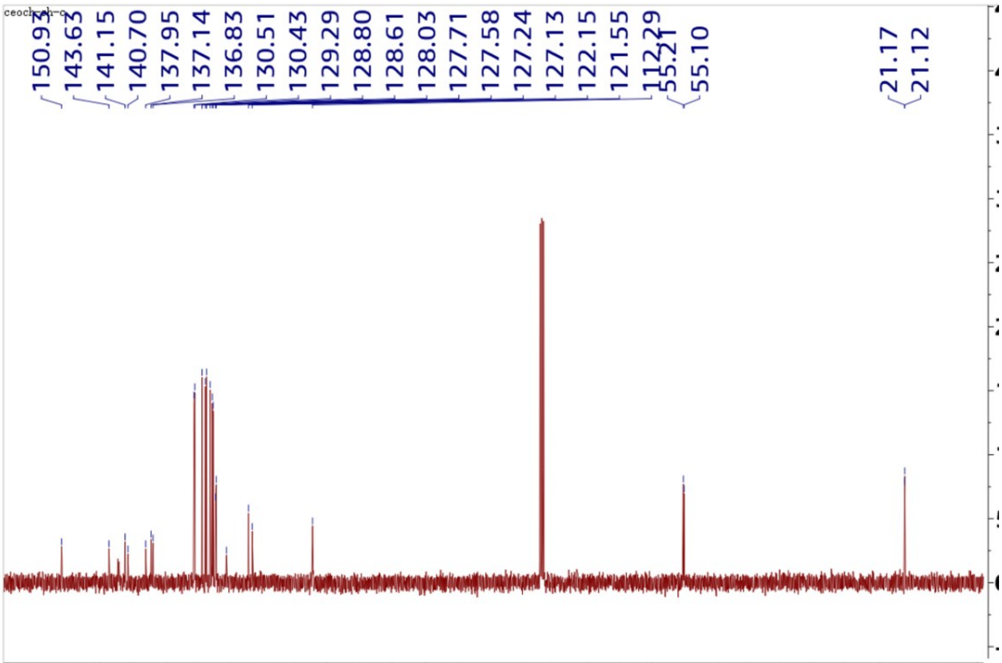

150.93
143.65
141.15
140.70
137.95
137.14
136.83
130.51
130.43
129.29
128.80
128.61
128.03
127.71
127.58
127.24
127.13
122.15
121.55
112.29
55.21
55.10
21.17
21.12
150
130
110
90
80
70
60
50
40
30
20
10
f1 (ppm)

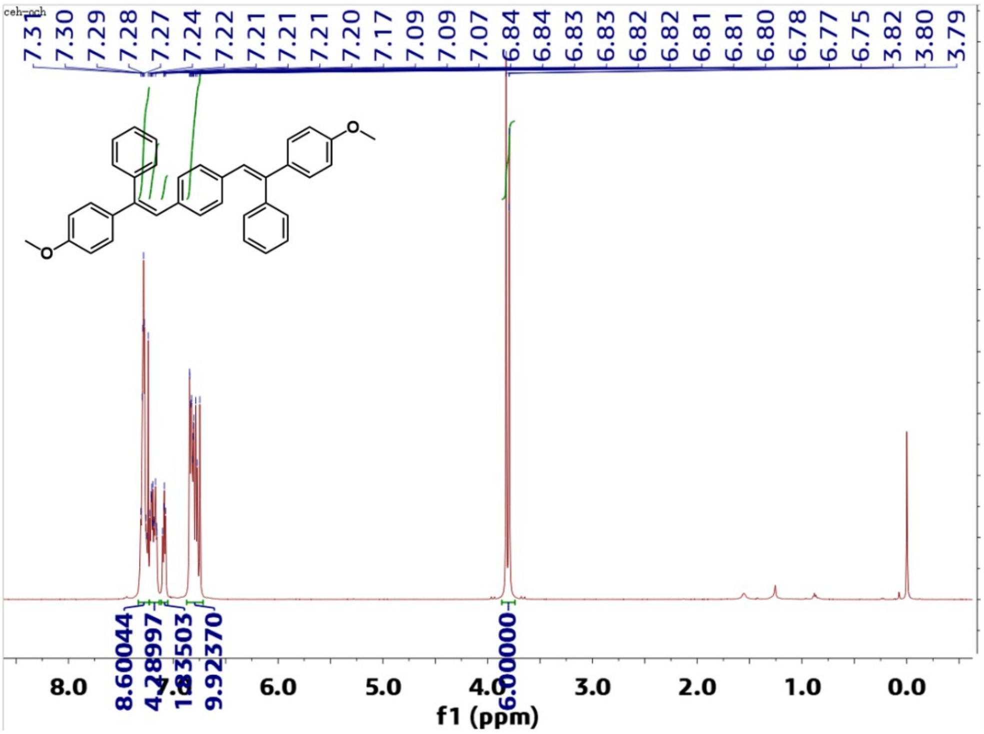

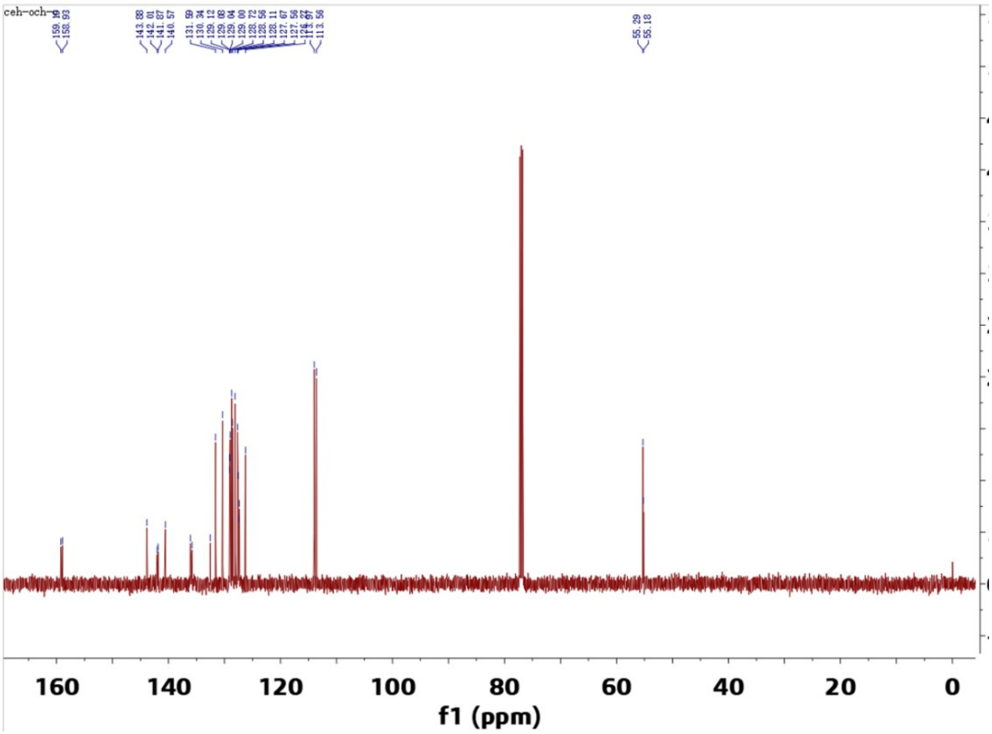

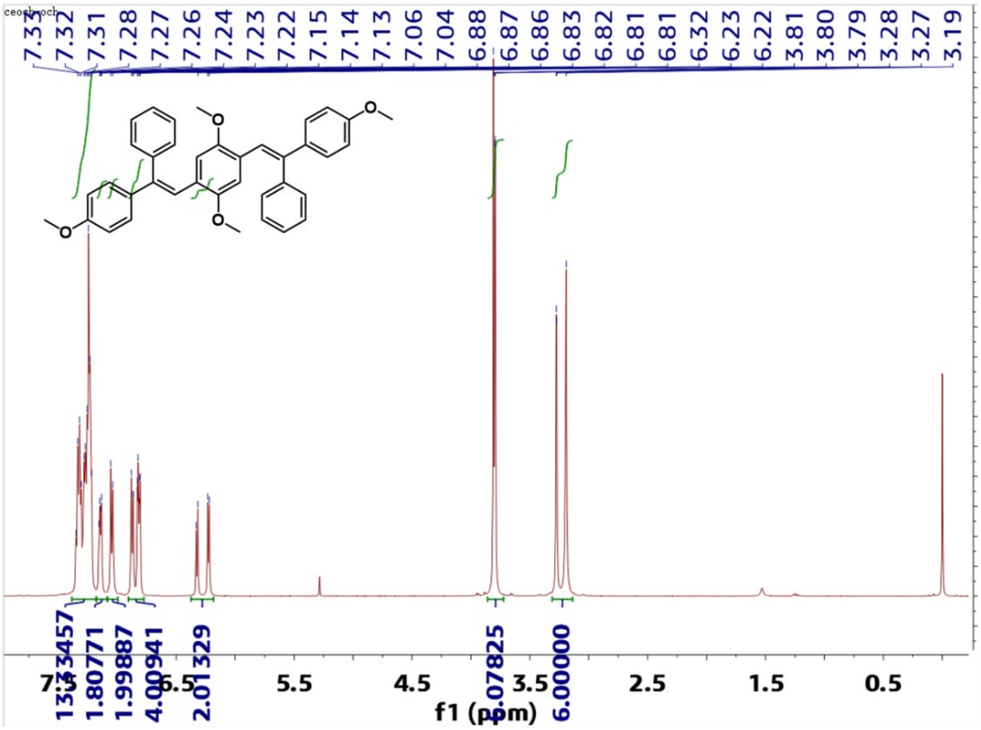
ceoch-och
7.33
7.32
7.31
7.28
7.27
7.26
7.24
7.23
7.22
7.15
7.14
7.13
7.06
7.04
6.88
6.87
6.86
6.83
6.82
6.81
6.81
6.32
6.23
6.22
3.81
3.80
3.79
3.28
3.27
3.19
13.33457
1.80771
1.99887
4.00941
2.01329
6.07825
6.00000
7.5
6.5
5.5
4.5
3.5
2.5
1.5
0.5
f1 (ppm)

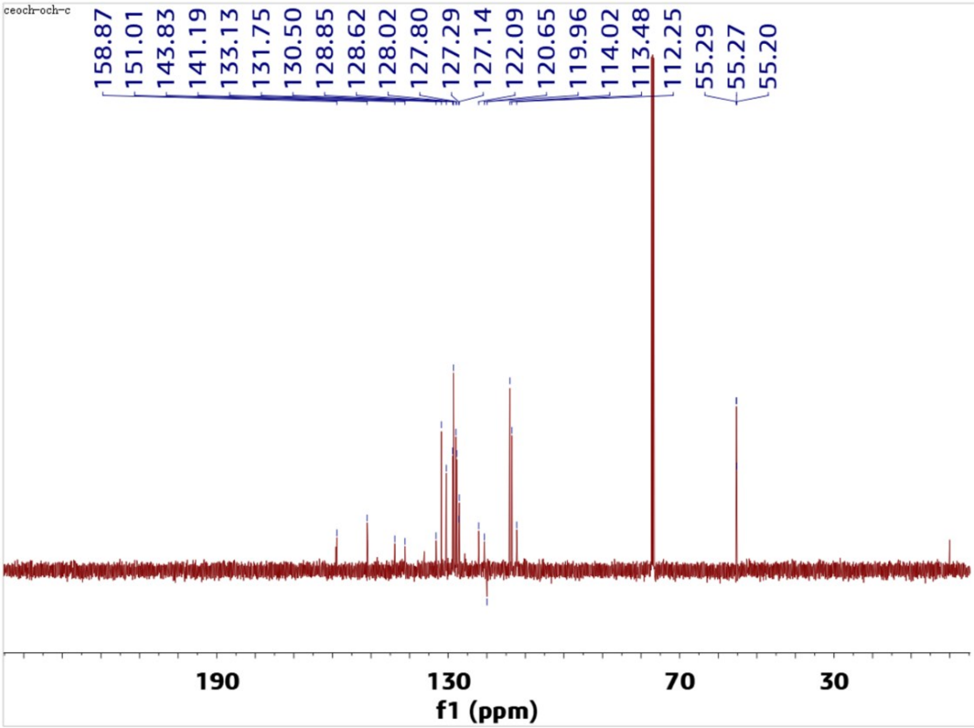
ceoch-och-c
158.87
151.01
143.83
141.19
133.13
131.75
130.50
128.85
128.62
128.02
127.80
127.29
127.14
122.09
120.65
119.96
114.02
113.48
112.25
55.29
55.27
55.20
190
130
70
30
f1 (ppm)

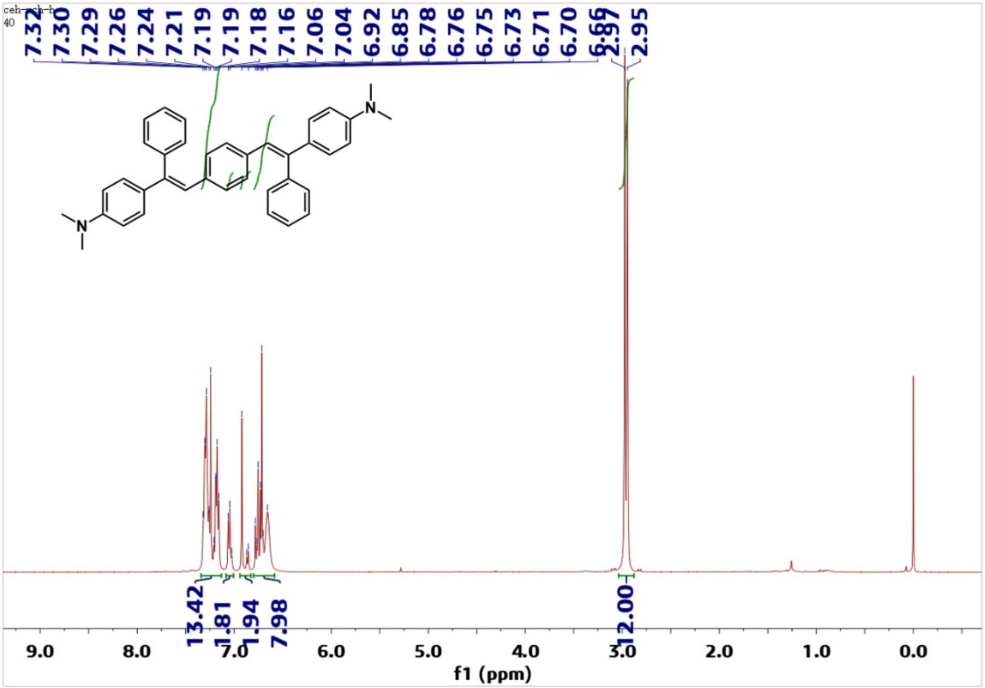
7.32
7.30
7.29
7.26
7.24
7.21
7.19
7.19
7.18
7.16
7.06
7.04
6.92
6.85
6.78
6.76
6.75
6.73
6.71
6.70
6.66
2.97
2.95
13.42
1.81
1.94
7.98
12.00
9.0
8.0
7.0
6.0
5.0
4.0
3.0
2.0
1.0
0.0
f1 (ppm)

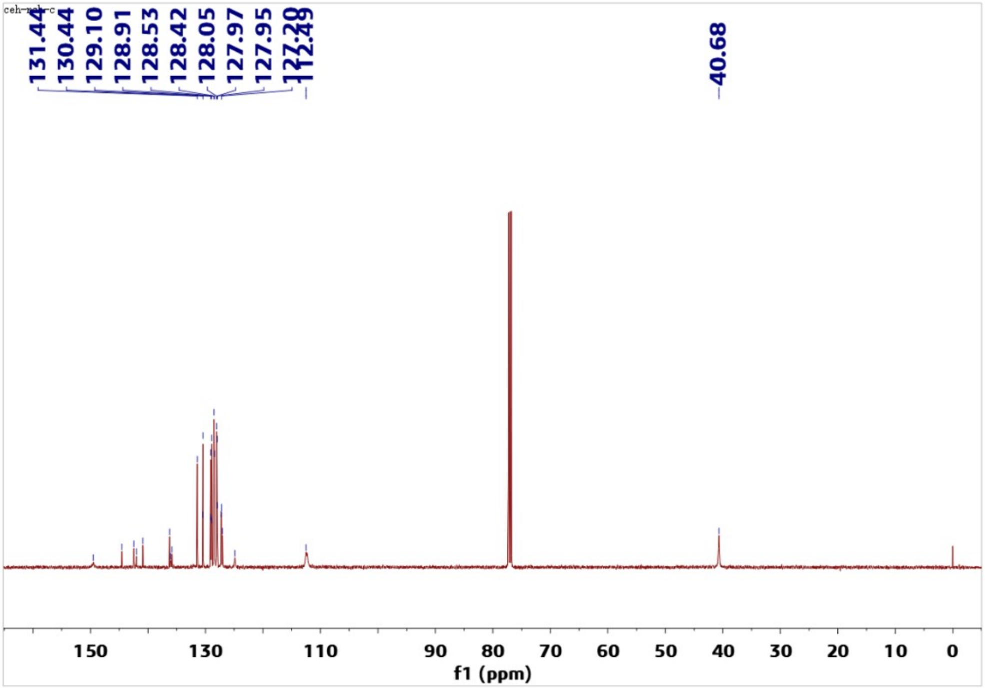
131.44
130.44
129.10
128.91
128.53
128.42
128.05
127.97
127.95
127.20
112.49
40.68
150
130
110
90
80
70
60
50
40
30
20
10
0
f1 (ppm)

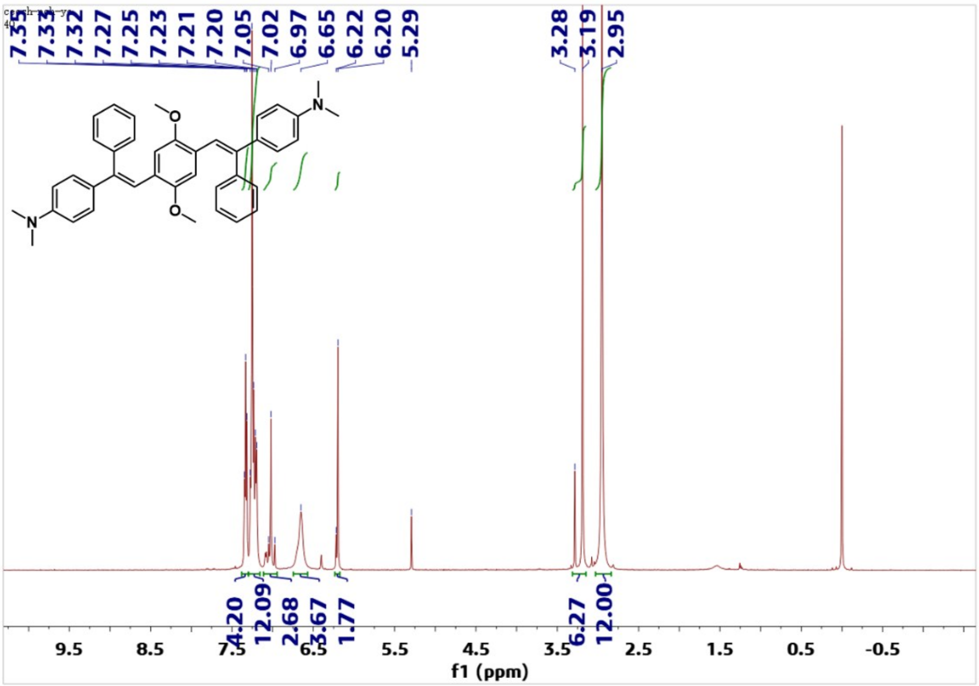
7.35 7.33 7.32 7.27 7.25 7.23 7.21 7.20 7.05 7.02 6.97 6.65 6.22 6.20 5.29
3.28 3.19 2.95
4.20 12.09 2.68 3.67 1.77 6.27 12.00
9.5 8.5 7.5 6.5 5.5 4.5 3.5 2.5 1.5 0.5 -0.5
f1 (ppm)

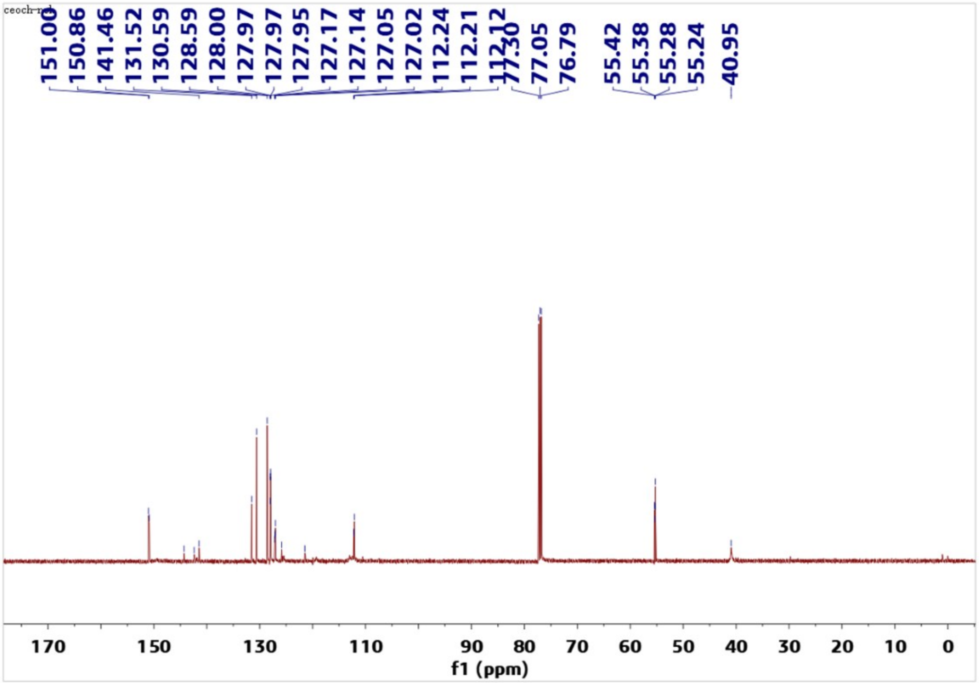
151.00 150.86 141.46 131.52 130.59 128.59 128.00 127.97 127.97 127.95 127.17 127.14 127.05 127.02 112.24 112.21 112.12 77.30 77.05 76.79 55.42 55.38 55.28 55.24 40.95
170 150 130 110 90 80 70 60 50 40 30 20 10 0
f1 (ppm)

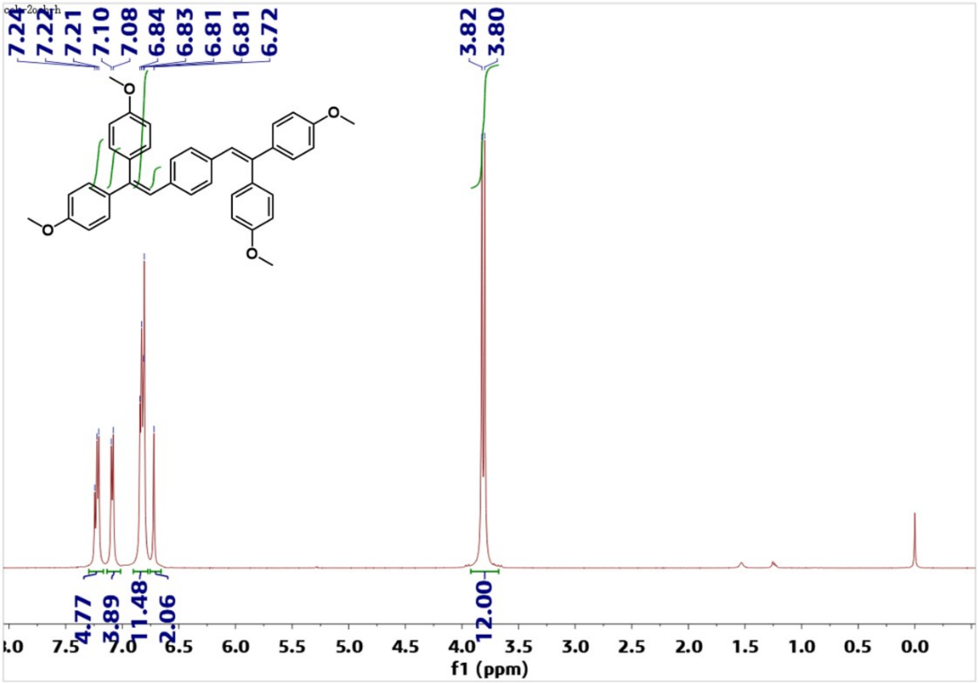

ceh-2och-h
7.24
7.22
7.21
7.10
7.08
6.84
6.83
6.81
6.81
6.72
3.82
3.80
4.77
3.89
11.48
2.06
12.00
8.0
7.5
7.0
6.5
6.0
5.5
5.0
4.5
4.0
3.5
3.0
2.5
2.0
1.5
1.0
0.5
0.0
f1 (ppm)

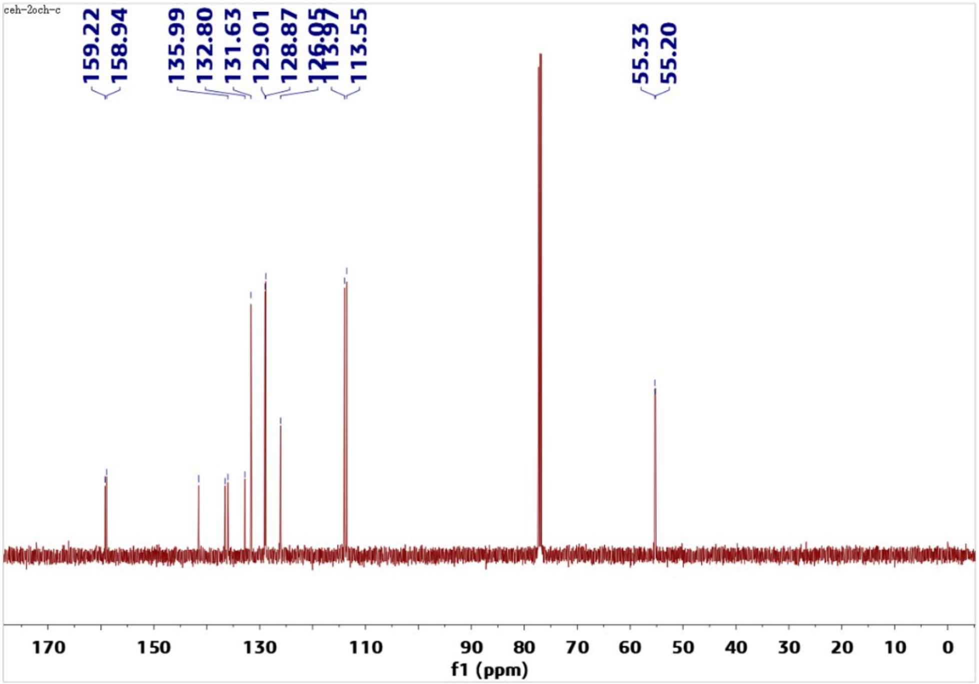

ceh-2och-c
159.22
158.94
135.99
132.80
131.63
129.01
128.87
126.05
113.97
113.55
55.33
55.20
170
150
130
110
90
80
70
60
50
40
30
20
10
0
f1 (ppm)

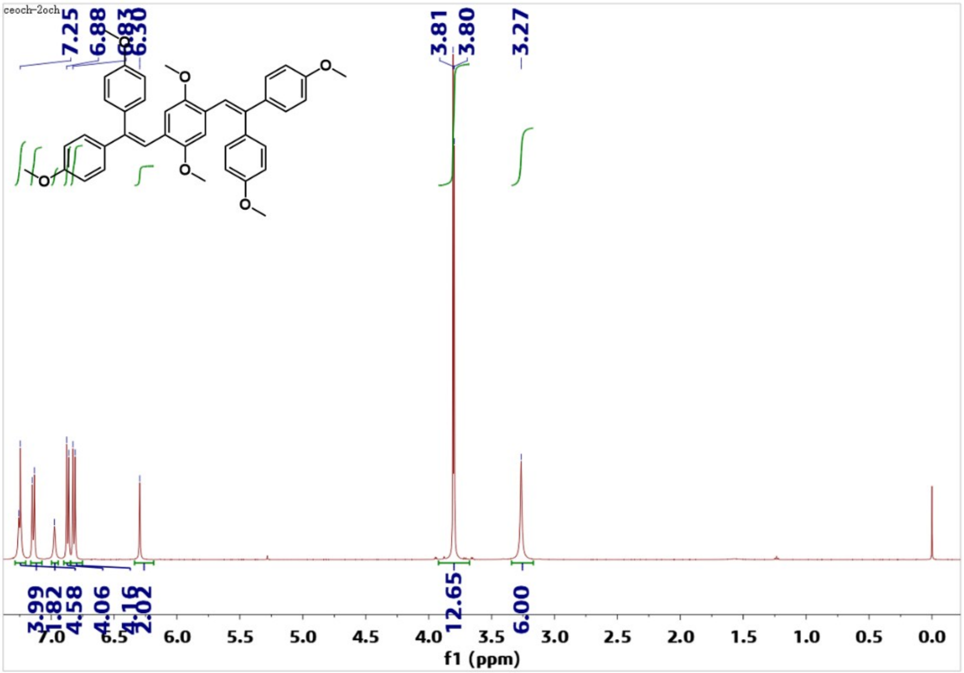
ceoch-2och
7.25
6.88
6.83
6.30
3.81
3.80
3.27
3.99
1.82
4.58
4.06
4.16
2.02
12.65
6.00
7.0 6.5 6.0 5.5 5.0 4.5 4.0 3.5 3.0 2.5 2.0 1.5 1.0 0.5 0.0
f1 (ppm)

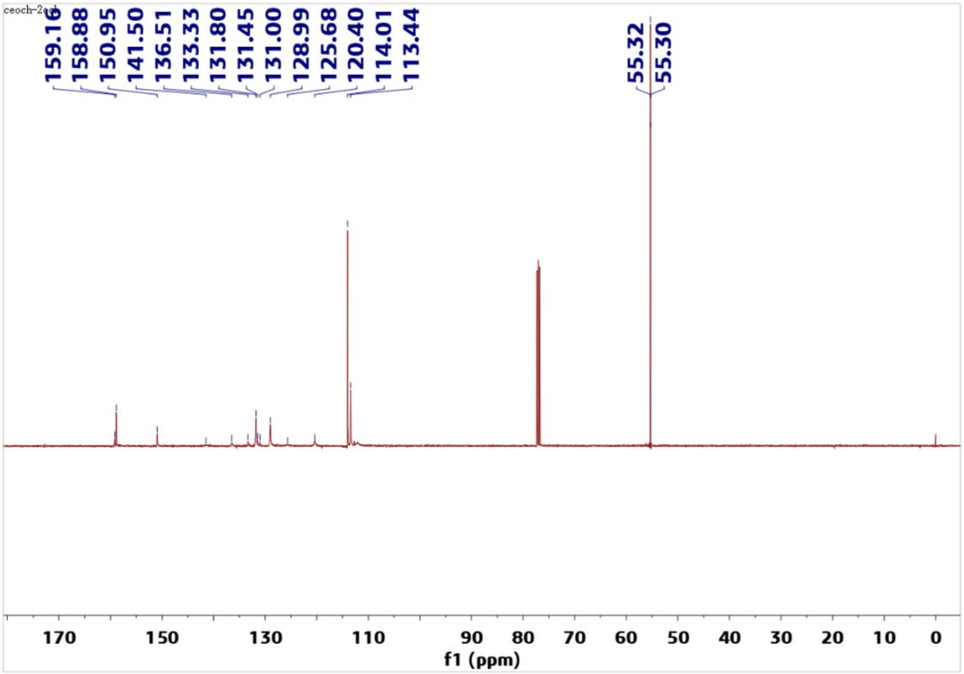
ceoch-2och
159.16
158.88
150.95
141.50
136.51
133.33
131.80
131.45
131.00
128.99
125.68
120.40
114.01
113.44
55.32
55.30
170 150 130 110 90 80 70 60 50 40 30 20 10 0
f1 (ppm)

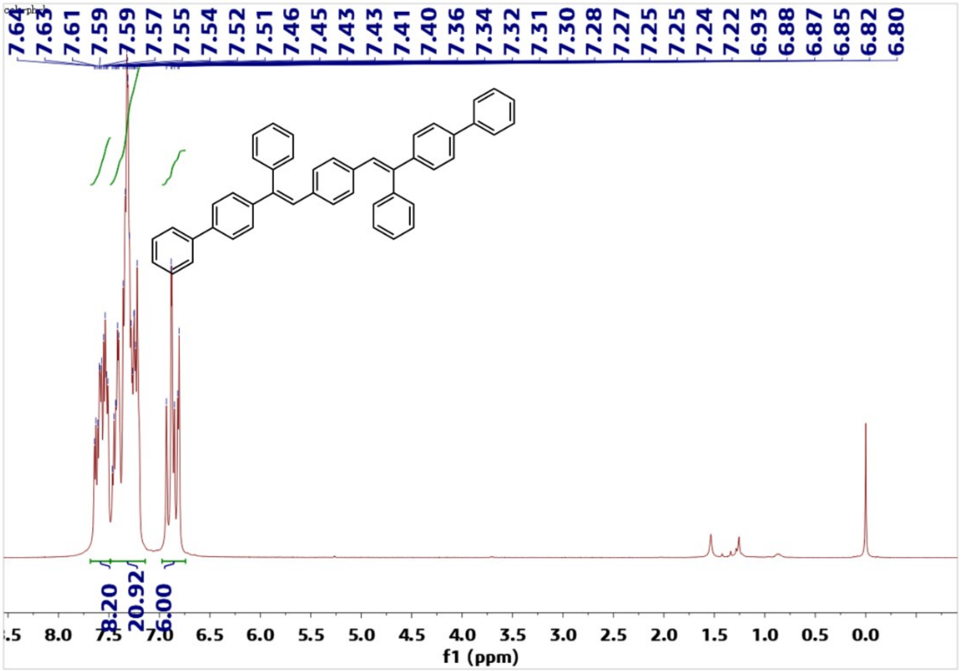
7.64 7.63 7.61 7.59 7.59 7.57 7.55 7.54 7.52 7.51 7.46 7.45 7.43 7.43 7.41 7.40 7.36 7.34 7.32 7.31 7.30 7.28 7.27 7.25 7.25 7.24 7.22 6.93 6.88 6.87 6.85 6.82 6.80
8.20
20.92
6.00
8.0 7.5 7.0 6.5 6.0 5.5 5.0 4.5 4.0 3.5 3.0 2.5 2.0 1.5 1.0 0.5 0.0
f1 (ppm)

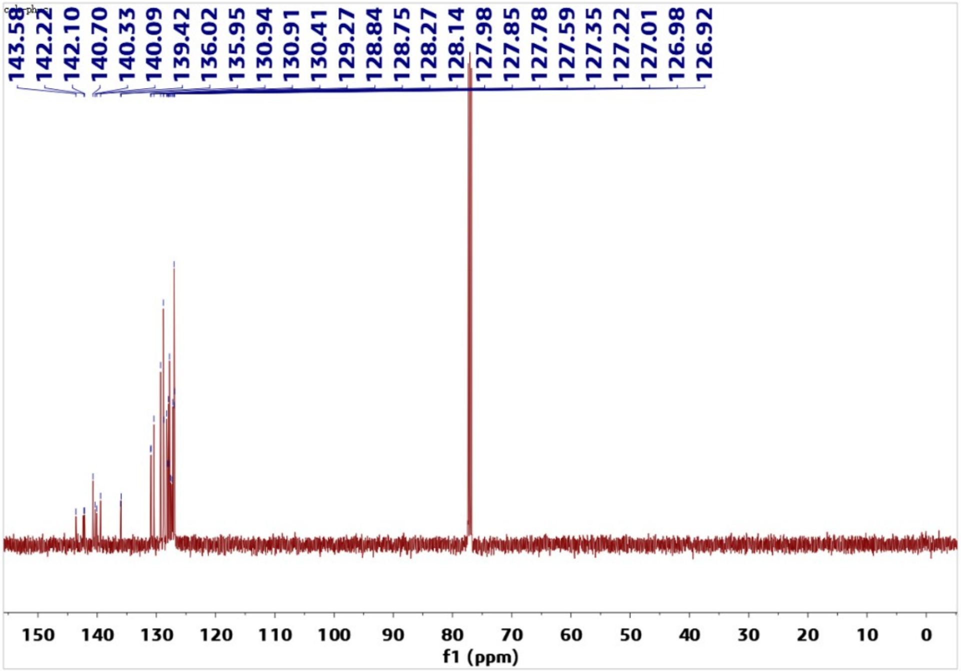
143.58 142.22 142.10 140.70 140.33 140.09 139.42 136.02 135.95 130.94 130.91 130.41 129.27 128.84 128.75 128.27 128.14 127.98 127.85 127.78 127.59 127.35 127.22 127.01 126.98 126.92
150 140 130 120 110 100 90 80 70 60 50 40 30 20 10 0
f1 (ppm)

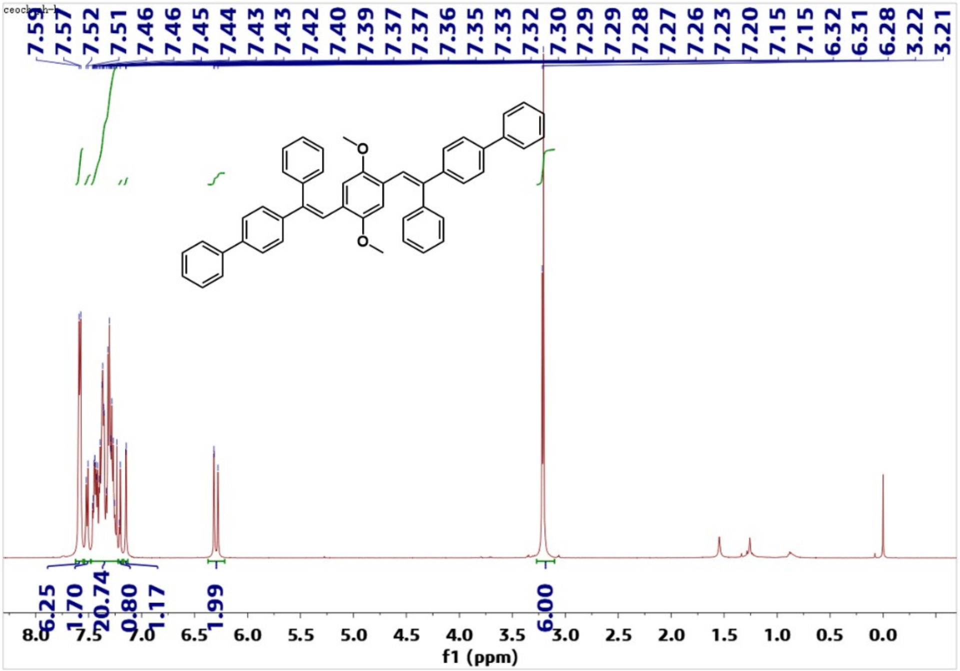
7.59 7.57 7.52 7.51 7.46 7.46 7.45 7.44 7.43 7.43 7.42 7.40 7.39 7.37 7.37 7.36 7.35 7.33 7.32 7.30 7.29 7.29 7.28 7.27 7.26 7.23 7.20 7.15 7.15 6.32 6.31 6.28 3.22 3.21
6.25 1.70 20.74 0.80 1.17 1.99 6.00
8.0 7.5 7.0 6.5 6.0 5.5 5.0 4.5 4.0 3.5 3.0 2.5 2.0 1.5 1.0 0.5 0.0
f1 (ppm)

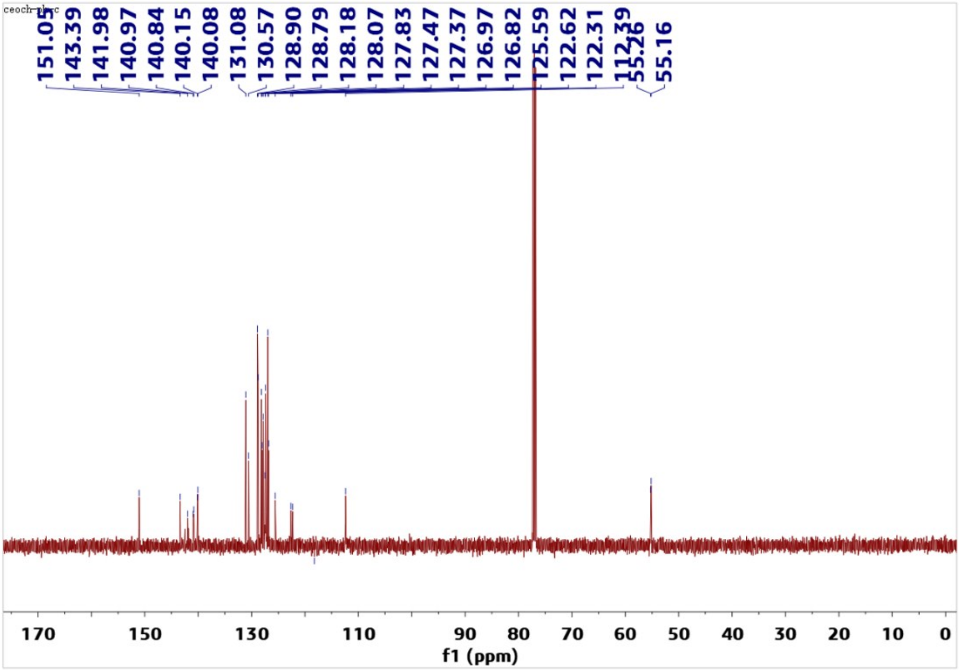
151.05 143.39 141.98 140.97 140.84 140.15 140.08 131.08 130.57 128.90 128.79 128.18 128.07 127.83 127.47 127.37 126.97 126.82 125.59 122.62 122.31 112.39 55.26 55.16
170 150 130 110 90 80 70 60 50 40 30 20 10 0
f1 (ppm)

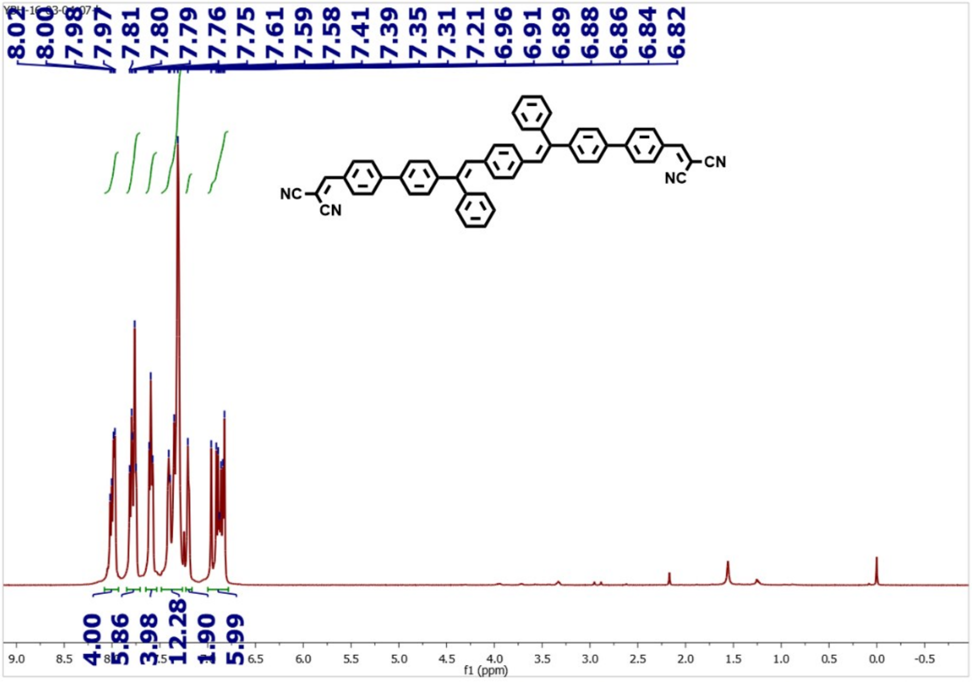
8.02 8.00 7.98 7.97 7.81 7.80 7.79 7.76 7.75 7.61 7.59 7.58 7.41 7.39 7.35 7.31 7.21 6.96 6.91 6.89 6.88 6.86 6.84 6.82
NC
CN
NC
CN
4.00 5.86 3.98 12.28 1.90 5.99
9.0 8.5 6.5 6.0 5.5 5.0 4.5 4.0 3.5 3.0 2.5 2.0 1.5 1.0 0.5 0.0 -0.5
f1 (ppm)

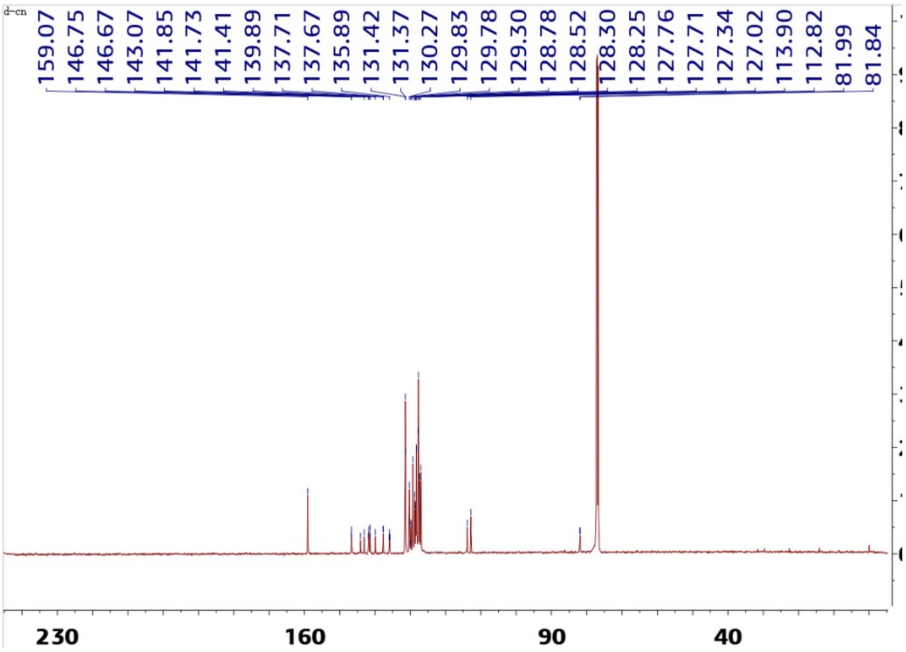
d-cn
159.07 146.75 146.67 143.07 141.85 141.73 141.41 139.89 137.71 137.67 135.89 131.42 131.37 130.27 129.83 129.78 129.30 128.78 128.52 128.30 128.25 127.76 127.71 127.34 127.02 113.90 112.82 81.99 81.84
230
160
90
40

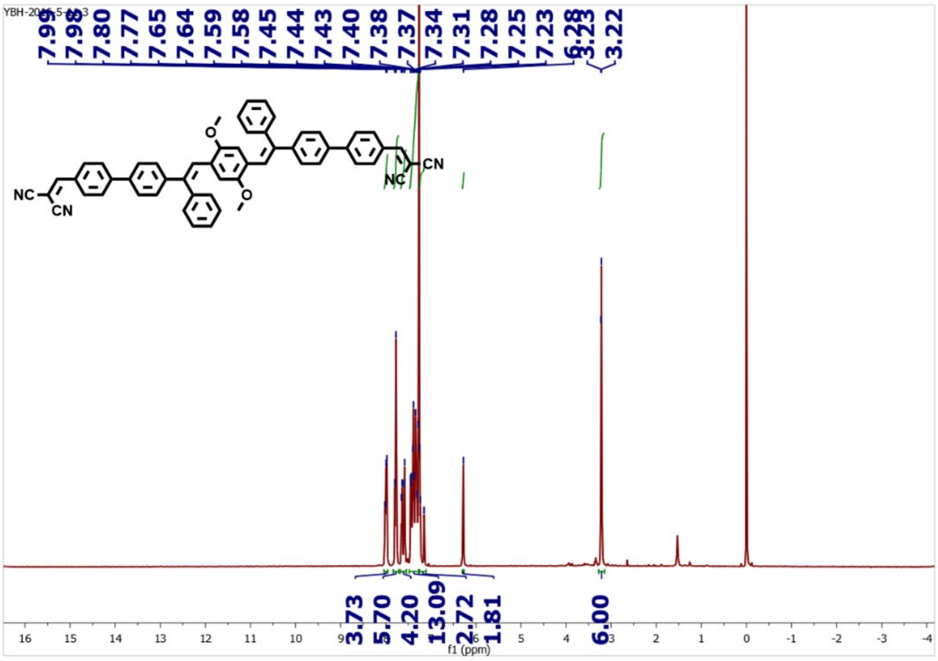

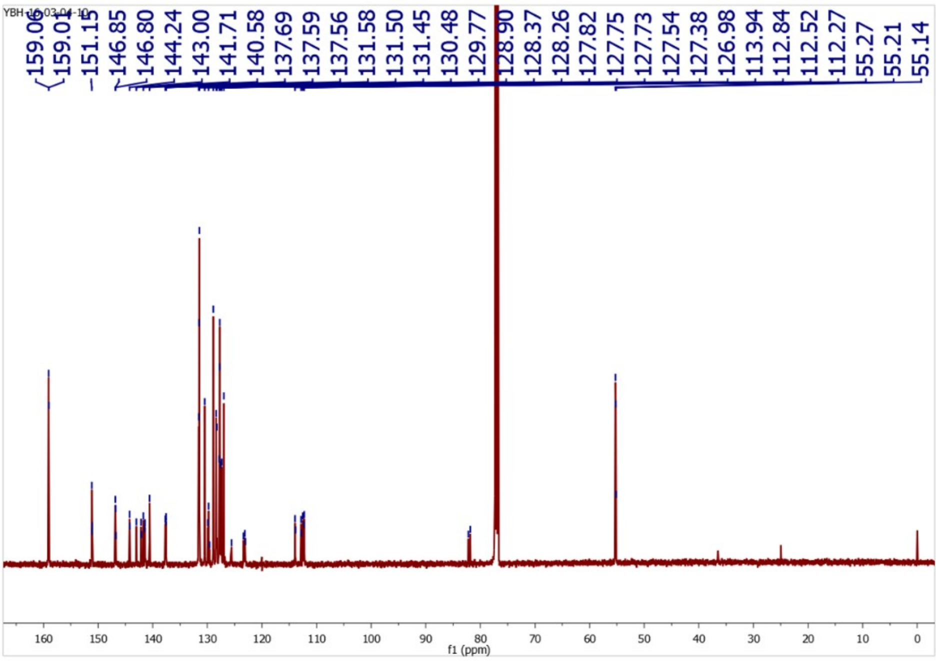

References

1 B. Yu, D. Liu, Y. Wang, T. Zhang, Y. M. Zhang, M. Li and S. X. Zhang, *Phys Chem Chem Phys*, 2018, **20**, 23851-23855.

2 B. Yu, D. Liu, Y. Wang, T. Zhang, Y.-M. Zhang, M. Li and S. X.-A. Zhang, Dyes Pigments, 2019, 163, 412-419.

3 Gaussian 09, Revision D.01,M. J. Frisch, G. W. Trucks, H. B. Schlegel, G. E. Scuseria, M. A. Robb, J. R. Cheeseman, G. Scalmani, V. Barone, B. Mennucci, G. A. Petersson, H. Nakatsuji, M. Caricato, X. Li, H. P. Hratchian, A. F. Izmaylov, J. Bloino, G. Zheng, J. L. Sonnenberg, M. Hada, M. Ehara, K. Toyota, R. Fukuda, J. Hasegawa, M. Ishida, T. Nakajima, Y. Honda, O. Kitao, H. Nakai, T. Vreven, J. A. Montgomery, Jr., J. E. Peralta, F. Ogliaro, M. Bearpark, J. J. Heyd, E. Brothers, K. N. Kudin, V. N. Staroverov, T. Keith, R. Kobayashi, J. Normand, K. Raghavachari, A. Rendell, J. C. Burant, S. S. Iyengar, J. Tomasi, M. Cossi, N. Rega, J. M. Millam, M. Klene, J. E. Knox, J. B. Cross, V. Bakken, C. Adamo, J. Jaramillo, R. Gomperts, R. E. Stratmann, O. Yazyev, A. J. Austin, R. Cammi, C. Pomelli, J. W. Ochterski, R. L. Martin, K. Morokuma, V. G. Zakrzewski, G. A. Voth, P. Salvador, J. J. Dannenberg, S. Dapprich, A. D. Daniels, O. Farkas, J. B. Foresman, J. V. Ortiz, J. Cioslowski, and D. J. Fox, Gaussian, Inc., Wallingford CT, 2013.

4 M. Brandl, M. S. Weiss, A. Jabs, J. Sühnel and R. Hilgenfeld, J. Mol. Biol., 2001, 307, 357.

5 H. Nie, K. Hu, Y. Cai, Q. Peng, Z. Zhao, R. Hu, J. Chen, S.-J. Su, A. Qin and B. Z. Tang, *Materials Chemistry Frontiers*, 2017, **1**, 1125-1129.
6 Q. Wu, C. Deng, Q. Peng, Y. Niu and Z. Shuai, *J Comput Chem*, 2012, **33**, 1862-1869.
7 T. Zhang, Y. Jiang, Y. Niu, D. Wang, Q. Peng and Z. Shuai, *J Phys Chem A*, 2014, **118**, 9094-9104.
8 Y. Xie, T. Zhang, Z. Li, Q. Peng, Y. Yi and Z. Shuai, *Chem Asian J*, 2015, **10**, 2154-2161.
